# A fast-linear mixed model for genome-wide haplotype association analysis: application to agronomic traits in maize

**DOI:** 10.1186/s12864-020-6552-x

**Published:** 2020-02-11

**Authors:** Heli Chen, Zhiyu Hao, Yunfeng Zhao, Runqing Yang

**Affiliations:** 1 Research Center for Aquatic Biotechnology, Chinese Academy of Fishery Sciences, Beijing, 100141 People’s Republic of China; 2 College of Animal Science and Technology, Northeast Agricultural University, Harbin, 150030 China

**Keywords:** GWAS, Linear mixed model, R/fastLmPure, Genomic heritability, Haplotype, Maize

## Abstract

**Background:**

Haplotypes combine the effects of several single nucleotide polymorphisms (SNPs) with high linkage disequilibrium, which benefit the genome-wide association analysis (GWAS). In the haplotype association analysis, both haplotype alleles and blocks are tested. Haplotype alleles can be inferred with the same statistics as SNPs in the linear mixed model, while blocks require the formulation of unified statistics to fit different genetic units, such as SNPs, haplotypes, and copy number variations.

**Results:**

Based on the FaST-LMM, the fastLmPure function in the R/RcppArmadillo package has been introduced to speed up genome-wide regression scans by a re-weighted least square estimation. When large or highly significant blocks are tested based on EMMAX, the genome-wide haplotype association analysis takes only one to two rounds of genome-wide regression scans. With a genomic dataset of 541,595 SNPs from 513 maize inbred lines, 90,770 haplotype blocks were constructed across the whole genome, and three types of markers (SNPs, haplotype alleles, and haplotype blocks) were genome-widely associated with 17 agronomic traits in maize using the software developed here.

**Conclusions:**

Two SNPs were identified for LNAE, four haplotype alleles for TMAL, LNAE, CD, and DTH, and only three blocks reached the significant level for TMAL, CD, and KNPR. Compared to the R/lm function, the computational time was reduced by ~ 10–15 times.

## Background

In genome-wide association studies (GWAS), single nucleotide polymorphisms (SNPs) are the smallest genetic units analyzed. Large genetic units can be obtained through the combination of multiple SNPs in different forms. For instance, haplotype blocks in high linkage disequilibrium [1–3], copy number variations (CNVs) [4, 5] in the form of repeated DNA sequences variation, and larger genetic units, including genes and gene sets (pathway) [6–8] are comprehensively annotated with the development of whole-genome DNA re-sequencing. Genome-wide association analysis for large genetic units shows major advantages over SNPs in relation to: 1) explaining large percentages of phenotype variations by the combined effects of multiple SNPs and 2) facilitating the study of mechanisms related to complex traits by biologically meaningful genetic units such as genes and pathways [9].

Using random polygenic effects excluding the tested marker to correct confounding factors, such as population stratification and cryptic relatedness, linear mixed models (LMM) improve the power to detect quantitative trait nucleotides (QTNs) by efficiently controlling false positive rates. However, the high computing intensity of LMM has motivated the development of simpler algorithms [10–17] to reduce the computational burden, allowing LMM to become a widely used and powerful approach in genome-wide association studies (GWAS). These simplified methods work by reducing the LMM or replacing the restricted maximum likelihood (REML) [18] with spectral decomposition. Although the reduced LMMs, such as GRAMMAR [10], EMMAX [11] or P3D [12], CMLM [12], GRAMMAR-Gamma [13], and BOLT-LMM [14], retain the same statistical power as the regular LMM, they over-estimate the residual polygenic effects and decrease the goodness-of-fit of phenotypes. Instead of REML, the efficient mixed-model association (EMMA) [15] avoids a redundant and computationally expensive matrix operation at each iteration in the computation of the likelihood function by the spectral decomposition of phenotype and marker indicators. As such, the computational speed to solve the LMM is substantially increased by several orders of magnitude. On the other hand, unlike EMMA (which spectrally decomposes each tested SNP), the factored spectrally transformed linear mixed model (FaST-LMM) [16] only requires a single spectral decomposition to test all SNPs, thereby offering a decrease in the memory footprint and additional speedups. Finally, the second derivatives for the log-likelihood function are considered in the genome-wide efficient mixed-model association (GEMMA) [17] algorithm, specifically based on the spectral decomposition, in order to determine the global optimum.

Based on the FaST-LMM [16], we transform the genome-wide mixed model association analysis to a linear regression scan, along with searching for variance components, and extend the FaST-LMM for SNPs to different genetic units by constructing a unified test statistic. To speed up genome-wide regression scans, we introduce the fastLmPure function in the R/RcppArmadillo package to infer the effect of tested genetic units. When only large or highly significant blocks obtained from EMMAX are tested, the genome-wide haplotype association analysis will reduce the analysis to one or two rounds of genome-wide regression scans. The software Single-RunKing [19] was developed to implement the extremely fast genome-wide mixed model association analysis for different genetic units. The high-computing efficiency of the software is demonstrated by the re-analyzing of 17 agronomic traits from the maize genomic datasets [20].

## Results

### Haplotype construction

**Fig. 1:**
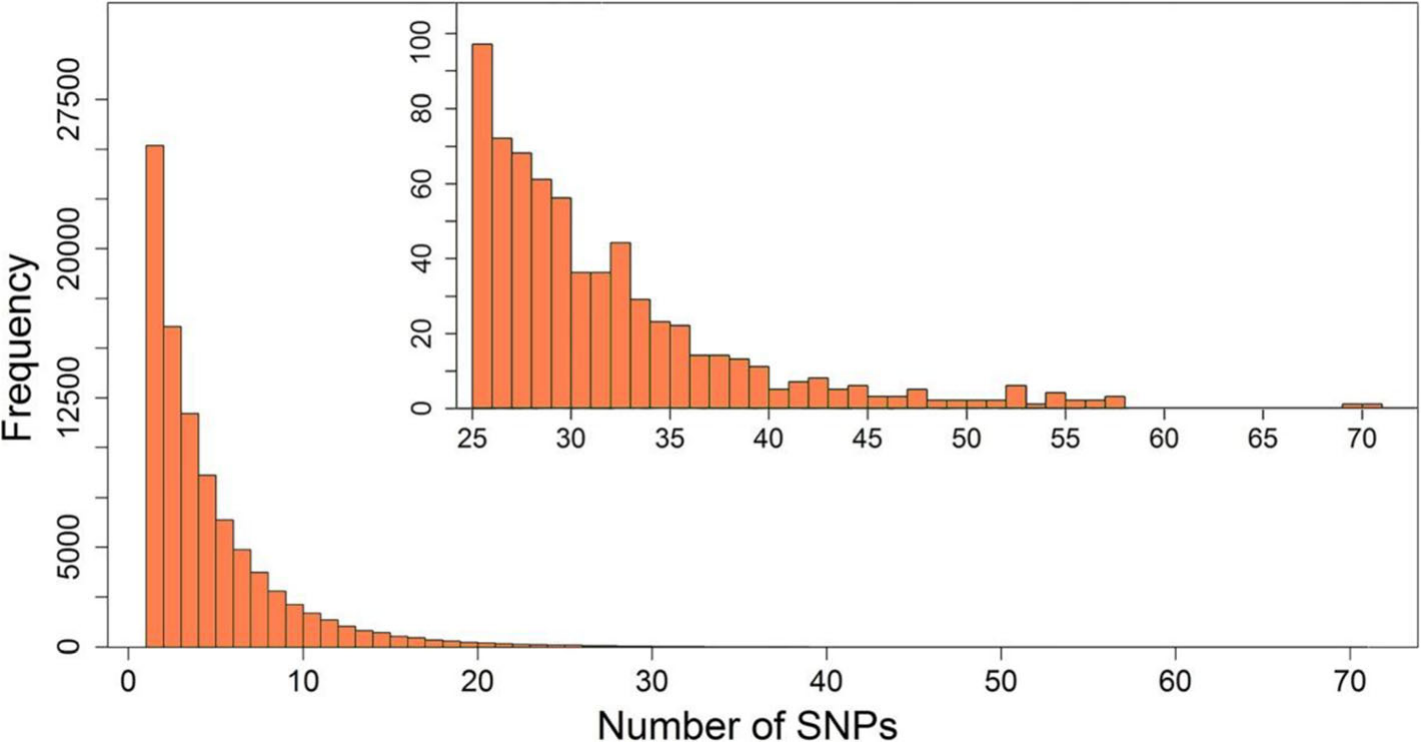
Distribution in numbers of SNPs forming haplotype blocks. The inner picture is an enlargement of the horizontal coordinates from 25 to 70

**Fig. 2:**
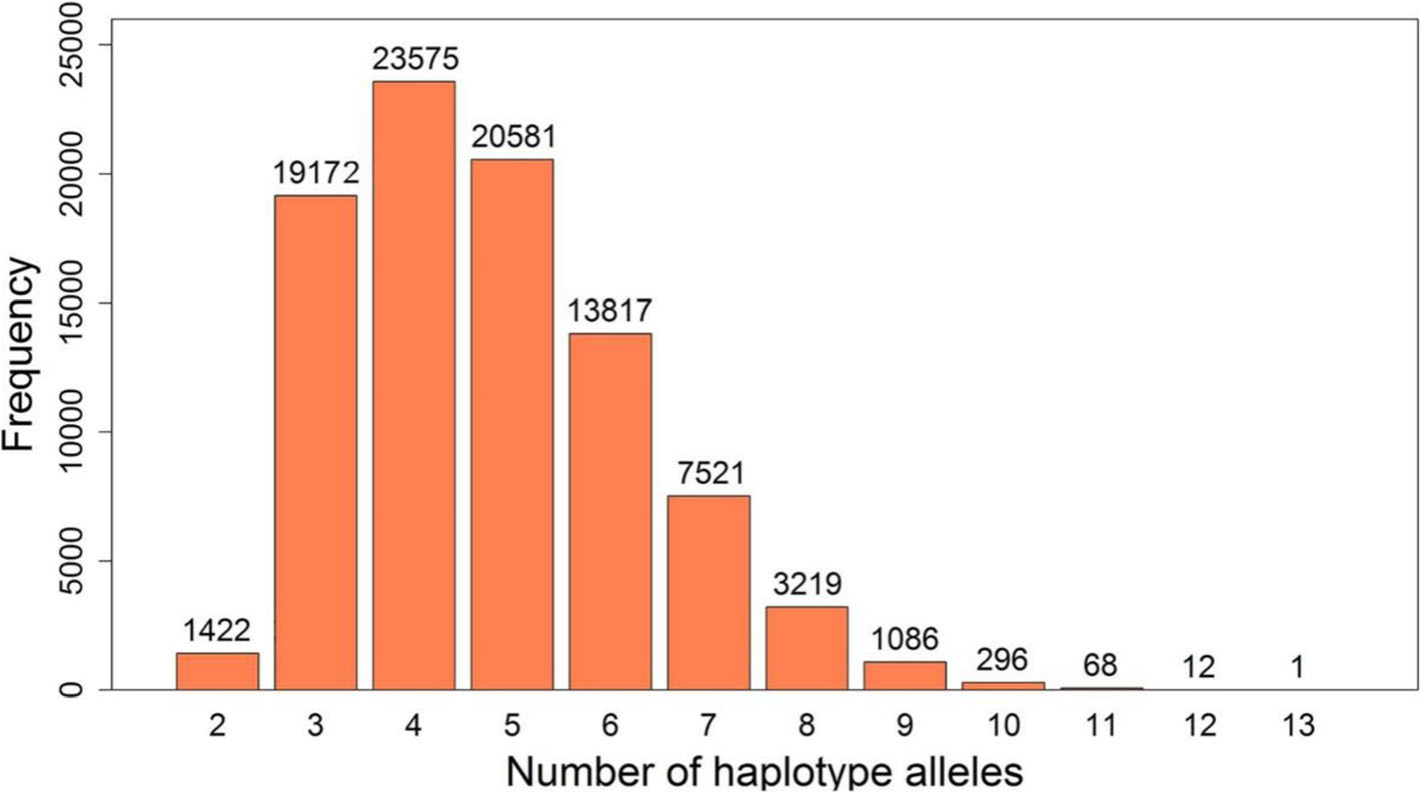
Distribution in number of haplotype alleles included in haplotype blocks

Haplotype blocks of the genomic dataset were constructed using the Four Gamete Test method (FGT) [21], which is implemented in the Haploview software [22]. With a cutoff of 1%, a total of 90,770 haplotype blocks were generated, covering 482,858 SNPs that account for 89.2% of all analyzed SNPs. Considering the number of SNPs included in each block, there were 59 kinds of blocks formed by more than 2 SNPs. Figure 1 displays the frequency of haplotype blocks that consist of different numbers of SNPs. More than 90% of the haplotype blocks contained less than 10 SNPs, with the largest block containing 71 SNPs. The number of haplotype alleles are less than the theoretical values in most blocks. Moreover, rare haplotype alleles with frequencies of less than 0.02 were merged to one allele in each block, so that only 432,505 haplotype alleles were collected. Figure 2 shows the distribution of the number of haplotype alleles included in the blocks, of which 85% of haplotype blocks yielded 3~6 alleles and the most haplotype alleles were 13 in a single block.

### GWAS for genetic units

We applied the Single-RunKing software to associate SNPs, haplotype alleles, and haplotype blocks genome-widely with 17 agronomic traits. Prior to GWAS, the two analyzed variables, SNPs and haplotype alleles, were assigned values 0 and 1, but the former corresponds to two homogeneous genotypes in the resource population and the latter depends on whether they occur in individuals. When haplotype blocks were analyzed, their last haplotype alleles were removed to make the regression of the block identifiable. At a significance level of 5%, the critical thresholds by the Bonferroni correction were determined as 7.035, 6.937, and 6.259 to declare significance for SNPs, haplotype alleles, and blocks, respectively. The agronomic traits were all associated with genome-wide SNPs, haplotype alleles, and blocks using the LM with unified test statistics and the Single-RunKing software based on the FaST-LMM.

All analyses were performed on a CentOS 6.5 operating system running in a server with a 2.60 GHz Intel Xeon E5–2660 Opteron (tm) Processor, 512 GB RAM, and 20 TB HDD. The data input took 8.7250, 9.0520, and 13.7064 min for haplotype blocks, haplotype alleles and SNPs, respectively, and preparation of input variables 3.4972, 3.4321, and 4.3497 min. More specifically, the Single-RunKing for the haplotype blocks, haplotype alleles, and SNPs consumed bare-bone regression scans of 1.6072, 3.7589, and 5.1181 min, respectively, which were significantly lower than that of the linear model implemented in the R/lm function (17.2284, 40.2937 and 54.8637 min). If only the SNPs with statistical probabilities of more than 0.05 were optimized, then the running time for bare-bone regression scans would reduce to 0.4527, 1.5235, and 1.6927 min using the Single-RunKing.

**Fig. 3:**
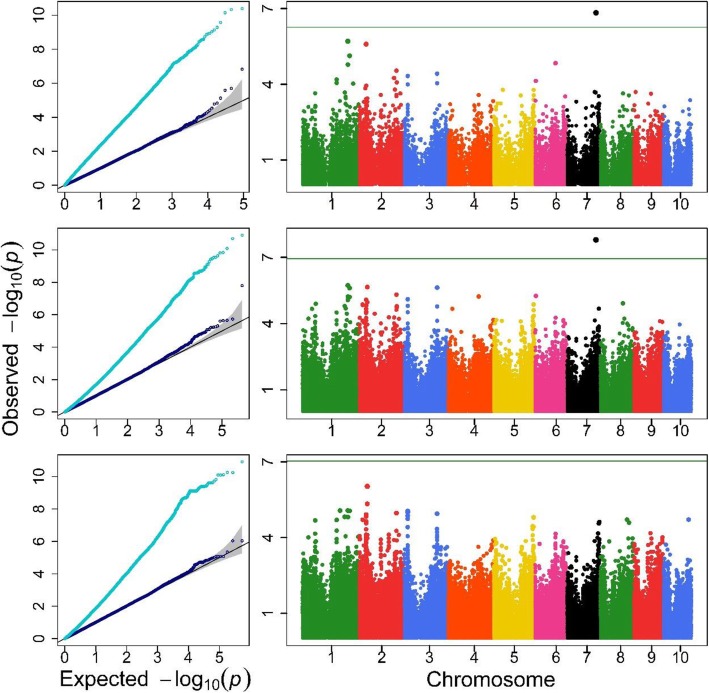
QQ and Manhattan plots of three genetic units for TMAL trait. The top, the medium and the bottom are for haplotype blocks, haplotype alleles and SNPs, respectively

**Fig. 4:**
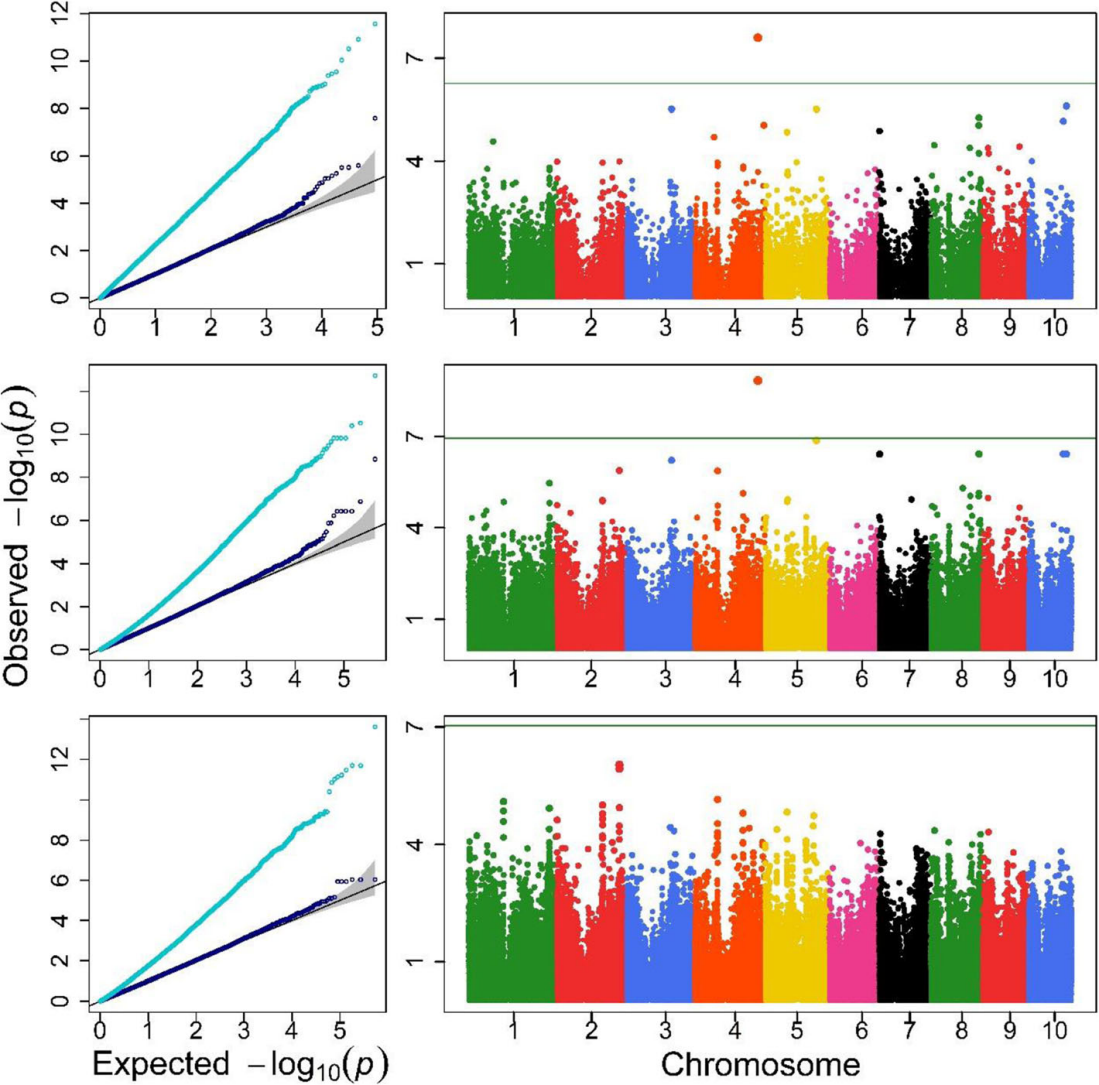
QQ and Manhattan plots of three genetic units for CD trait. The top, the medium and the bottom are for haplotype blocks, haplotype alleles and SNPs, respectively

**Fig. 5:**
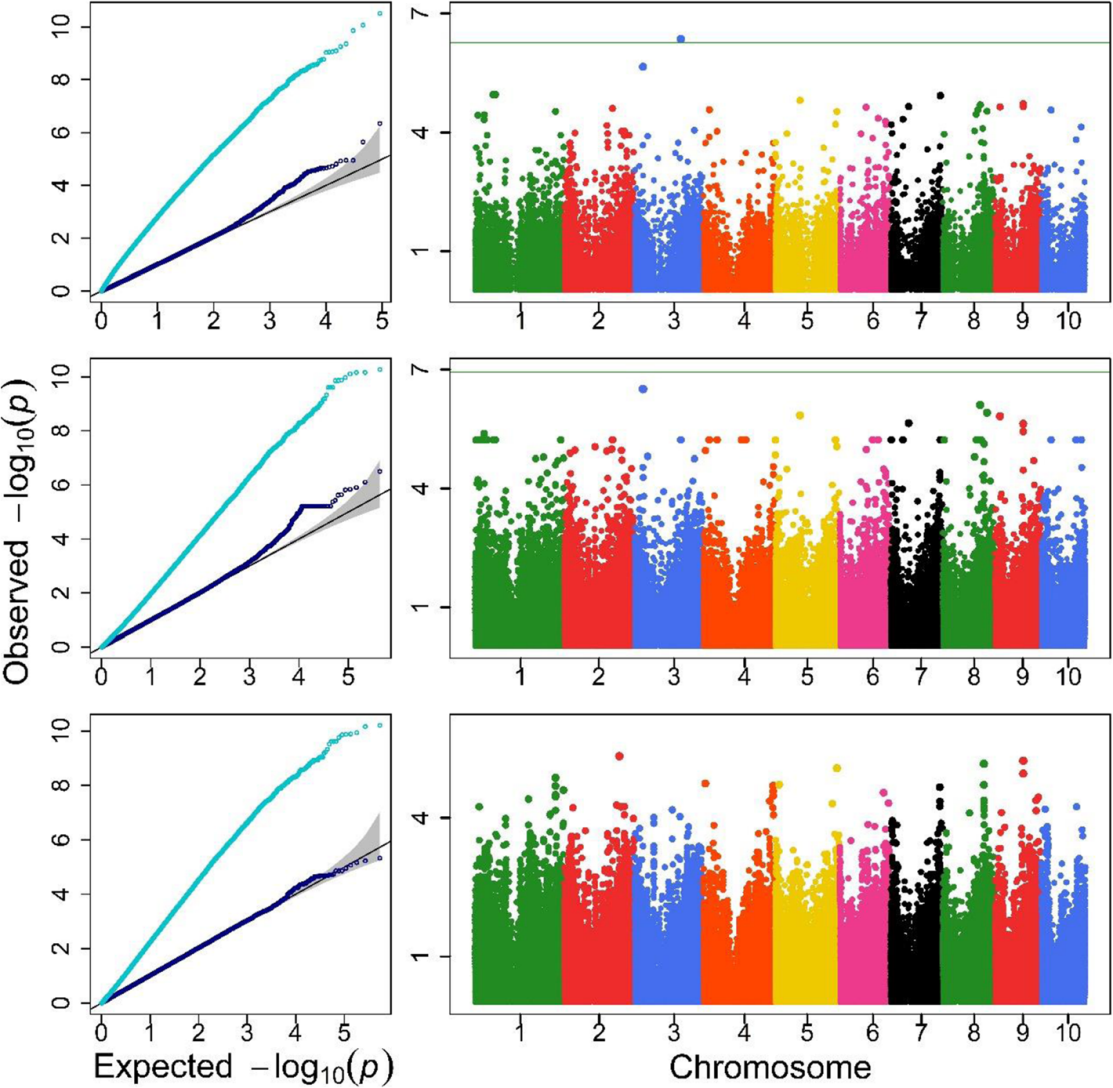
QQ and Manhattan plots of three genetic units for KNPR trait. The top, the medium and the bottom are for haplotype blocks, haplotype alleles and SNPs, respectively

Q-Q and Manhattan plots are depicted in Fig. 3, 4 and 5 and Additional file 1: Figure S1-S2 for the agronomic traits with detected QTLs. In each Q-Q plot obtained with the Single-RunKing software, the real line for –log_10_(*p*) nearly overlaps with the theoretical expectation except for the high end of the line, and the genomic control values were closed to 1 (see Additional file 1: Table S1). This suggests that, compared to the LM algorithm, which seriously inflates test statistics, the Single-RunKing software performs excellent genomic controls for the confounding factors. According to the Manhattan plots, GWAS using the Single-RunKing software are summarized in Table 1 for the agronomic traits. At least one type of genetic unit was identified for only five traits: TMAL, LNAE, CD, KNPR, and DTH. No SNPs, haplotype alleles, and blocks were located together for the same trait, with two types of genetic units at most being located for a specific trait. Only two SNPs (chr4.S_216,248,578 and chr4.S_216,248,611), which are in high degree of linkage disequilibrium, were detected for LNAE, with the haplotype allele Chr4Block6251_2 (where they reside) being also significant. Two haplotype alleles and their corresponding blocks were simultaneously found to significantly control TMAL and CD, respectively. Only one block, Chr3Block4589, was detected for KNPR, while one haplotype allele, Chr3Block7921_rare, was detected for DTH. The two detectable SNPs, chr4.S_216,248,578 and chr4.S_216,248,611, explained 7.33 and 7.38% of the phenotypic variation, respectively. The four haplotype alleles accounted for 0.54 to 10.16% of the phenotypic variation, while the three haplotype blocks accounted for 1.98, 6.64, and 10.69%, which are quite larger than the corresponding SNPs or haplotype alleles detected. Additionally, all the detected genetic units were mapped on the annotated genes, especially Chr3Block4589 on two genes with known biological meaning.

**Table 1:** Three types of significant genetic units identified for 17 traits using the Single-RunKing software

Traits	QTL	Chr	Position (bp)	haplotypes	SNPs	−log_10_(*p*)	Heritability (%)	Candidate gene
TMAL	Block5106	7	154,269,126~154,269,135	4	2	6.83*	1.98	GRMZM5G835323
	Block5106_rare					7.79*	0.54	
LNAE	Block6251	4	216,248,578~216,248,659	4	3	6.00		GRMZM2G138881
	Block6251_2					7.32*	7.43	
	chr4.S_216,248,578		216,248,578			7.29*	7.33	
	chr4.S_216,248,611		216,248,611			7.18*	7.38	
CD	Block6253	4	216,318,748~216,319,308	5	3	7.60*	10.69	GRMZM2G477205
	Block6253_rare					8.84*	10.16	
KNPR	Block4589	3	156,814,466~156,936,687	4	2	6.35*	6.64	GRMZM2G336909 GRMZM2G089952
DTH	Block7921	3	211,147,258~211,147,654	6	7	5.47		GRMZM2G422651
	Block7921_rare					7.45*	7.62	

## Discussion

Using spectral decomposition of phenotypes and markers, the FaST-LMM transformed the LMM of the tested marker to LM. Genetic effects of markers were estimated with re-weighted least square, along with optimization of genomic variance. A unified test statistic was formulated to fit different genetic units, such as SNPs, haplotypes, and copy number variations. In GWAS implemented in the Single-RunKing software, computational efficiency is greatly improved in three ways: 1) by using the bare-bones linear model fitting function, known as R/fastLmPure, to rapidly estimate genetic effects of the tested SNPs, 2) by replacing genomic variance with heritability to narrow down the search of solutions, and 3) by focusing on large or highly significant SNPs obtained with EMMAX. The Single-RunKing software was developed to transform the genome-wide mixed model association analysis into bare-bones regression scans, where the optimal polygenic heritability of the tested markers is searched by the re-weighted least square estimation of the genetic effects. Given the genomic heritability, the EMMAX method needs a genome-wide regression scan of only one round. Based on the EMMAX method, the Single-RunKing software will run genome-wide regression scans within two rounds if only large or highly significant markers are tested.

In genome-wide mixed model association analysis, the construction of kinship matrix by all markers will consume increasingly more memory footprint and computing time, given that more high-throughput SNPs are produced by re-sequencing techniques. Furthermore, the computing time required would be incredibly high if the kinship matrices vary with the tested markers. Counterproductively, the use of all or too many SNPs to calculate kinship matrices may yield proximal contamination [16, 23, 24] due to the over-estimation of polygenic variance, especially for large genetic units. The simplest approach is to use random samples of genetic markers to construct the kinship matrices [12, 24]. Selectively including and/or excluding pseudo QTNs to derive kinship matrices for the tested SNPs can improve statistical power compared to deriving overall kinship matrices from all or a random sample of genetic markers [23, 25]. Additionally, the CMLM reduces the dimension of the RRM by clustering individuals into several groups based on the selected genetic markers. If the resource population is too large, a random sample of the population can also be used to rapidly estimate genomic heritability. Overall, in order to improve computing efficiency, all simplified procedures of the genome-wide mixed model association analysis can be incorporated into the Single-RunKing software.

In real data analysis, the genetic units SNP, haplotype alleles, and blocks were analyzed, of which the former is included in the latter. As produced with the analysis of variance, three possible outcomes were detected among the three genetic units: the first which consists of both the former and the latter, the second which is only the former or only the latter, and the third is neither the former nor the latter. With respect to the five mapped traits, three mapping outcomes occurred between haplotype alleles and corresponding blocks. Only one significant SNP was identified together with one corresponding haplotype allele for LNAE. In our test, among the four significant haplotype alleles, three were merged by rare alleles with low frequency in one block. After being applied for the genome-wide mixed model association analysis, the haplotype blocks explained more phenotypic variation than the detected corresponding SNPs or haplotype alleles due to the combined effects of multiple SNPs.

## Conclusion

A bare-bones linear model fitting function, known as R/fastLmPure, was used to rapidly estimate effects of genetic units and maximum likelihood values of the FaST-LMM. When only large or highly significant genetic units are tested based on the EMMAX, the extended Single-RunKing software for genetic units takes genome-wide regression scans one to two times. The algorithm was applied into the genome-wide association of agronomic traits in maize. Three haplotype blocks were identified for TMAL, CD, and KNPR traits, while four haplotype alleles were found for TMAL, LNAE, CD, and DTH traits.

## Methods

### Maize genomic data

The dataset was downloaded from http://www.maizego.org/Resources.html. After a high-quality control was established, 541,595 SNPs for 508 maize inbred lines remained for the subsequent analysis. For constructing haplotypes, missing genotypes were imputed by BEAGLE [26]. The analyzed traits include plant height (PH), ear height (EH), ear leaf width (ELW), ear leaf length (ELL), tassel main axis length (TMAL), tassel branch number (TBN), leaf number above ear (LNAE), ear length (EL), ear diameter (ED), cob diameter (CD), kernel number per row (KNPR), 100-grain weight (GW), cob weight (CW), kernel width (KW), days to anthesis (DTA), days to silking (DTS), and days to heading (DTH).

### FaST-LMM for genetic units

In matrix notation, general LMM for GWAS can be described as:

$$ \mathbf{y}=\mathbf{1}\mu +\mathbf{X}\boldsymbol{\upbeta } +\mathbf{Za}+\boldsymbol{\upvarepsilon}, $$

where y is a vector of the phenotypic values from *n* individuals, which is justified for systemic factors that include population stratification; *μ* is the population mean; β is the additive genetic effect of the tested genetic units, such as the SNP, haplotype (or block), and copy number variations; **a** is a vector of *n* random polygenic effects excluding the genetic unit tested, which subjects to the distribution ${N}_n\left(\mathbf{0},\mathbf{K}{\sigma}_a^2\right)$ with a realized relationship matrix (RRM) [27–30] **K** calculated from genetic markers and an unknown polygenic variance ${\sigma}_a^2$; **ε** is a vector of *n* random residual effects, which are mutually independent among individuals and follow the distribution ${N}_n\left(\mathbf{0},\mathbf{I}{\sigma}_{\varepsilon}^2\right)$ with identity matrix **I** and residual variance ${\sigma}_{\varepsilon}^2$; **1** is a column vector of *n* orders; and **X** and **Z** are the incidence matrices for β and **a**, respectively.

The LMM satisfied:

$$ \mathrm{Var}\left(\mathbf{y}|\boldsymbol{\upbeta} \right)=\mathbf{K}{\sigma}_a^2+\mathbf{I}{\sigma}_{\varepsilon}^2. $$

With polygenic heritability ${h}^2={\sigma}_a^2/\left({\sigma}_a^2+{\sigma}_{\varepsilon}^2\right)$ replacing ${\sigma}_a^2$ [19], the covariance matrix becomes:

$$ \mathrm{Var}\left(\mathbf{y}|\boldsymbol{\upbeta} \right)=\left(\frac{h^2}{1-{h}^2}\mathbf{K}+\mathbf{I}\right){\sigma}_{\varepsilon}^2. $$

Following the FaST-LMM algorithm [16], we spectrally decompose **K** = **USU**^**T**^, where **S** is the diagonal matrix containing the eigenvalues of **K** in descending order, and **U** is the matrix of the eigenvectors corresponding to the eigenvalues. According to **UU**^**T**^ = **I**, the covariance matrix can be written as:

$$ \mathrm{Var}\left(\mathbf{y}|\boldsymbol{\upbeta} \right)=\mathbf{U}\left(\frac{h^2}{1-{h}^2}\mathbf{S}+\mathbf{I}\right){\mathbf{U}}^{\mathbf{T}}{\sigma}_{\varepsilon}^2. $$

Let $\tilde{\mathbf{y}}={\mathbf{U}}^{\mathbf{T}}\mathbf{y}$ and $\tilde{\mathbf{X}}={\mathbf{U}}^{\mathbf{T}}\left[\mathbf{1}\kern0.24em \mathbf{X}\right]$, after which the LMM is transformed to the following linear model (LM):

$$ \tilde{\mathbf{y}}=\tilde{\mathbf{X}}\boldsymbol{\upbeta} +\mathbf{e}, $$

where $\mathbf{e}\sim {N}_n\left(\mathbf{0},\mathbf{W}{\sigma}_{\varepsilon}^2\right)$ with $\mathbf{W}=\frac{h^2}{1-{h}^2}\mathbf{S}+\mathbf{I}$ as the diagonal matrix.

When genetic units such as haplotypes (or blocks) and CNVs can be divided into more than three genotypes, it is required that one of those genotypes is constricted to 0 to make the LM identifiable. With the weighted least square method, the maximum likelihood estimates of β and ${\sigma}_{\varepsilon}^2$ are obtained as follows:

$$ {\displaystyle \begin{array}{l}\hat{\boldsymbol{\upbeta}}={\left(\tilde{\mathbf{X}}{\mathbf{W}}^{-1}{\tilde{\mathbf{X}}}^{\mathbf{T}}\right)}^{-1}{\tilde{\mathbf{X}}}^{\mathbf{T}}{\mathbf{W}}^{-1}\tilde{\mathbf{y}}\\ {}{\hat{\sigma}}_{\varepsilon}^2=\frac{1}{n-1}{\left(\tilde{\mathbf{y}}-\tilde{\mathbf{X}}\hat{\boldsymbol{\upbeta}}\right)}^{\mathbf{T}}{\mathbf{W}}^{-1}\left(\tilde{\mathbf{y}}-\tilde{\mathbf{X}}\hat{\boldsymbol{\upbeta}}\right)\end{array}}. $$

With $\hat{\boldsymbol{\upbeta}}$ and ${\hat{\sigma}}_{\varepsilon}^2$, the maximum likelihood value of the LM is estimated as:

$$ L=\frac{1}{\sqrt{2\pi \mid \mathbf{W}{\hat{\sigma}}_{\varepsilon}^2\mid }}\exp \left[\frac{1}{{\hat{\sigma}}_{\varepsilon}^2}{\left(\tilde{\mathbf{y}}-\tilde{\mathbf{X}}\hat{\boldsymbol{\upbeta}}\right)}^{\mathbf{T}}{\mathbf{W}}^{-1}\left(\tilde{\mathbf{y}}-\tilde{\mathbf{X}}\hat{\boldsymbol{\upbeta}}\right)\right]. $$

The log-likelihood is further simplified as:

$$ -2\log L\propto n\log {\hat{\sigma}}_{\varepsilon}^2+\log \mid \mathbf{W}\mid, $$

which represents the polygenic heritability *h*^*2*^ in the weighted diagonal matrix W. Thus, we can optimize this function of *h*^2^ using a one-dimensional scan within the open interval (0, 1) to find the maximum likelihood estimate of *h*^2^. At the same time, the genetic effect of the tested genetic unit is statistically inferred by $\hat{\beta}$ and ${\hat{\sigma}}_{\varepsilon}^2$ corresponding to the optimized *h*^2^. The test statistic for the genetic unit is unified to:

$$ F=\frac{1}{d{f}_{\beta }{\hat{\sigma}}_{\varepsilon}^2}\left[{\left(\mathbf{y}-\mathbf{1}\mu \right)}^{\mathbf{T}}\left(\mathbf{y}-\mathbf{1}\mu \right)-d{f}_{\varepsilon }{\hat{\sigma}}_{\varepsilon}^2\right] $$

which subjects to the F distribution with degrees of freedom *df*_*β*_ as the number of genotypes in the tested genetic unit minus one (*df*_*ε*_ = *n* − *df*_*β*_ − 1), and *F* ∼ *t*(*df*_*β*_) in terms for testing SNPs. For a large sample, *F* ∼ *χ*^2^(*df*_*β*_) with *χ*^2^(1) is used for the SNP tested.

### Implementation

As stated earlier, the FaST-LMM [16] transforms the genome-wide mixed model association analysis into linear regression scans by re-weighted least square estimations for effects of genetic units, along with optimization of polygenic heritabilities. To speed up computational efficiency, the regression analysis for the tested genetic unit is implemented with the bare-bones linear model fitting function, known as fastLmPure, in the R/RcppArmadillo package [19]. The fastLmPure function in the R software runs dozens of times faster than the lm function. The fastLmPure function returns only the genetic effect and the standard error of the tested genetic unit, and statistics, such as ${\sigma}_{\varepsilon}^2$, −2logL, student *t*, and *p* value, need to be calculated after running the fastLmPure function.

In generating input variables, **y** and **X** have been spectrally transformed into **y’** and **X’**, respectively. Given polygenic heritability, the weighted diagonal matrix **W** is generated, and then the dependent variable (${\mathbf{y}}^{\ast }={\mathbf{W}}^{-\frac{1}{2}}\tilde{\mathbf{y}}$) and independent variable (${\mathbf{X}}^{\ast }={\mathbf{W}}^{-\frac{1}{2}}\tilde{\mathbf{X}}$) are calculated. Based on these variables, the subroutine to solve the LMM with the bare-bones regression is written as:

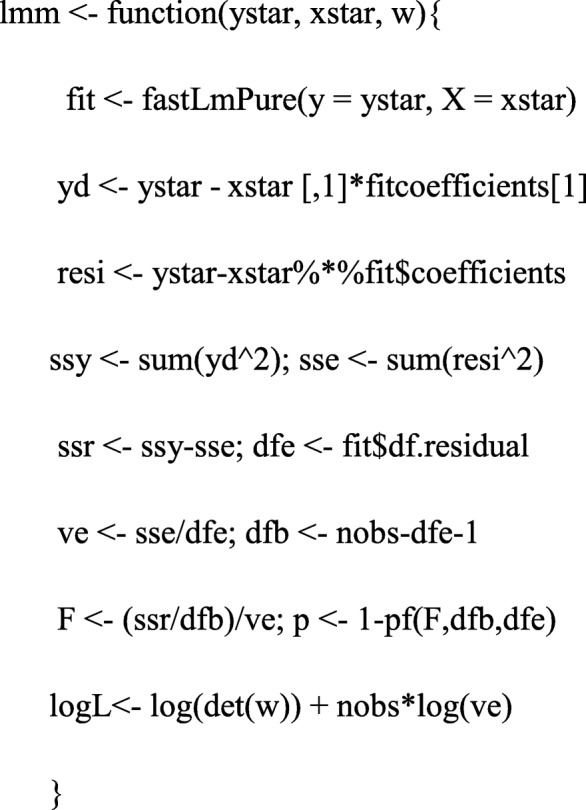



Theoretically, the polygenic heritability for the tested genetic unit is equal to the difference between the genomic heritability of traits and the genetic unit heritability (the proportion that explains the phenotypic variance by the genetic unit). Although polygenic heritabilities differ among high-throughput genetic units, they are very close to the genomic heritability of traits because most genetic units, except for QTLs, have no influence on quantitative traits. The genomic heritability of traits must be pre-estimated based on the LMM without the genetic unit effect. Starting from the estimated genomic heritability of quantitative traits, we can search downward to rapidly determine maximum likelihood estimates for the polygenic heritability of the tested genetic unit. Once the polygenic heritability for each genetic unit is fixed at a genomic heritability, the fast regression scan mentioned earlier is simplified as the EMMAX algorithm [11], of which its genome-wide scanning speed reaches the highest value using the fastLmPure function without optimization of polygenic heritabilities. This suggests that the genetic effects and statistical probabilities estimated by EMMAX qualify to serve as references for the fast regression scans for each genetic unit. To further enhance computing efficiency, we only selected genetic units of large effects or those with high significance levels (0.05 or 0.01) from the EMMAX algorithm to optimize the estimation of their polygenic heritabilities [19]. Thus, the computing time complexity for the genome-wide mixed model association analysis becomes *O* (*imn*) with *i* being the time of the genome-wide regression scans (1 < *i* ≤ 2). Based on this, the Single-RunKing software [19] written in R was extended to implement the genome-wide mixed model association analysis for genetic units in an extremely fast manner (see the codes in Additional file 1).

## Supplementary information

**Additional file 1: Table S1.** Genomic control values (GC) for the 5 traits with the QTLs detected by the Single-RunKing software. **Figure S1.** QQ and Manhattan plots of three genetic units for LNAE trait. The top, the medium and the bottom are for haplotype blocks, haplotype alleles and SNPs, respectively. **Figure S2.** QQ and Manhattan plots of three genetic units for DTH trait. The top, the medium and the bottom are for haplotype blocks, haplotype alleles and SNPs, respectively. **Figure S3.** QQ and Manhattan plots of three genetic units for PH trait. The top, the medium and the bottom are for haplotype blocks, haplotype alleles and SNPs, respectively. **Figure S4.** QQ and Manhattan plots of three genetic units for EH trait. The top, the medium and the bottom are for haplotype blocks, haplotype alleles and SNPs, respectively. **Figure S5.** QQ and Manhattan plots of three genetic units for ELW trait. The top, the medium and the bottom are for haplotype blocks, haplotype alleles and SNPs, respectively. **Figure S6.** QQ and Manhattan plots of three genetic units for ELL trait. The top, the medium and the bottom are for haplotype blocks, haplotype alleles and SNPs, respectively. **Figure S7.** QQ and Manhattan plots of three genetic units for TBN trait. The top, the medium and the bottom are for haplotype blocks, haplotype alleles and SNPs, respectively. **Figure S8.** QQ and Manhattan plots of three genetic units for EL trait. The top, the medium and the bottom are for haplotype blocks, haplotype alleles and SNPs, respectively. **Figure S9.** QQ and Manhattan plots of three genetic units for ED trait. The top, the medium and the bottom are for haplotype blocks, haplotype alleles and SNPs, respectively. **Figure S10.** QQ and Manhattan plots of three genetic units for GW trait. The top, the medium and the bottom are for haplotype blocks, haplotype alleles and SNPs, respectively. **Figure S11.** QQ and Manhattan plots of three genetic units for CW trait. The top, the medium and the bottom are for haplotype blocks, haplotype alleles and SNPs, respectively. **Figure S12.** QQ and Manhattan plots of three genetic units for KW trait. The top, the medium and the bottom are for haplotype blocks, haplotype alleles and SNPs, respectively. **Figure S13.** QQ and Manhattan plots of three genetic units for DTS trait. The top, the medium and the bottom are for haplotype blocks, haplotype alleles and SNPs, respectively. **Figure S14.** QQ and Manhattan plots of three genetic units for DTA trait. The top, the medium and the bottom are for haplotype blocks, haplotype alleles and SNPs, respectively.

## Data Availability

The datasets analyzed in the current study were free downloaded from http://www.maizego.org/Resources.html, where gene resequencing data was available under GenBank accession number: JX404032–JX405439.

## Abbreviations

CD: Cob diameter
CNV: Copy number variation
CW: Cob weight
DTA: Days to anthesis
DTH: Days to heading
DTS: Days to silking
ED: Ear diameter
EH: Ear height
EL: Ear length
ELL: Ear leaf length
ELW: Ear leaf width
EMMA: Efficient mixed-model association
FaST-LMM: Factored spectrally transformed linear mixed model
FGT: Four gamete test
GEMMA: Genome-wide efficient mixed-model association
GW: 100-grain weight
GWAS: Genome-wide association studies
KNPR: Kernel number per row
KW: Kernel width
LMM: Linear mixed models
LNAE: Leaf number above ear
PH: Plant height
QTN: Quantitative trait nucleotide
REML: Restricted maximum likelihood
RRM: Realized relationship matrix
SNP: Single nucleotide polymorphisms
TBN: Tassel branch number
TMAL: Tassel main axis length
