# Supplementary material for: A fast-linear mixed model for genome-wide haplotype association analysis: application to agronomic traits in maize
Source: BMC Genomics. 2020 Feb 11;21:151. doi: 10.1186/s12864-020-6552-x (PMC7014697; doi:10.1186/s12864-020-6552-x)
Supplement: Supplementary file 1 — Additional file 1: Table S1. Genomic control values (GC) for the 5 traits with the QTLs detected by the Single-RunKing software. Figure S1. QQ and Manhattan plots of three genetic units for LNAE trait. The top, the medium and the bottom are for haplotype blocks, haplotype alleles and SNPs, respectively. Figure S2. QQ and Manhattan plots of three genetic units for DTH trait. The top, the medium and the bottom are for haplotype blocks, haplotype alleles and SNPs, respectively. Figure S3. QQ and Manhattan plots of three genetic units for PH trait. The top, the medium and the bottom are for haplotype blocks, haplotype alleles and SNPs, respectively. Figure S4. QQ and Manhattan plots of three genetic units for EH trait. The top, the medium and the bottom are for haplotype blocks, haplotype alleles and SNPs, respectively. Figure S5. QQ and Manhattan plots of three genetic units for ELW trait. The top, the medium and the bottom are for haplotype blocks, haplotype alleles and SNPs, respectively. Figure S6. QQ and Manhattan plots of three genetic units for ELL trait. The top, the medium and the bottom are for haplotype blocks, haplotype alleles and SNPs, respectively. Figure S7. QQ and Manhattan plots of three genetic units for TBN trait. The top, the medium and the bottom are for haplotype blocks, haplotype alleles and SNPs, respectively. Figure S8. QQ and Manhattan plots of three genetic units for EL trait. The top, the medium and the bottom are for haplotype blocks, haplotype alleles and SNPs, respectively. Figure S9. QQ and Manhattan plots of three genetic units for ED trait. The top, the medium and the bottom are for haplotype blocks, haplotype alleles and SNPs, respectively. Figure S10. QQ and Manhattan plots of three genetic units for GW trait. The top, the medium and the bottom are for haplotype blocks, haplotype alleles and SNPs, respectively. Figure S11. QQ and Manhattan plots of three genetic units for CW trait. The top, the medium and the bottom are for haplotype [file 12864_2020_6552_MOESM1_ESM.docx]

**Table S1 Genomic control values (GC) for the 5 traits with the QTLs detected by the Single-RunKing software.**

| **Traits** | **Genetic units** | **Single-RunKing** | **R/lm** |
| --- | --- | --- | --- |
| TMAL | Blocks | 1.007 | 1.981 |
|  | Haplotypes | 1.011 | 2.009 |
|  | SNPs | 1.007 | 2.257 |
| LNAE | Blocks | 1.009 | 3.093 |
|  | Haplotypes | 1.010 | 3.147 |
|  | SNPs | 1.007 | 3.736 |
| CD | Blocks | 1.011 | 1.878 |
|  | Haplotypes | 1.013 | 1.895 |
|  | SNPs | 1.007 | 2.070 |
| KNPR | Blocks | 1.005 | 2.345 |
|  | Haplotypes | 1.003 | 2.399 |
|  | SNPs | 1.012 | 2.807 |
| DTH | Blocks | 1.013 | 4.320 |
|  | Haplotypes | 1.014 | 4.385 |
|  | SNPs | 1.009 | 5.412 |


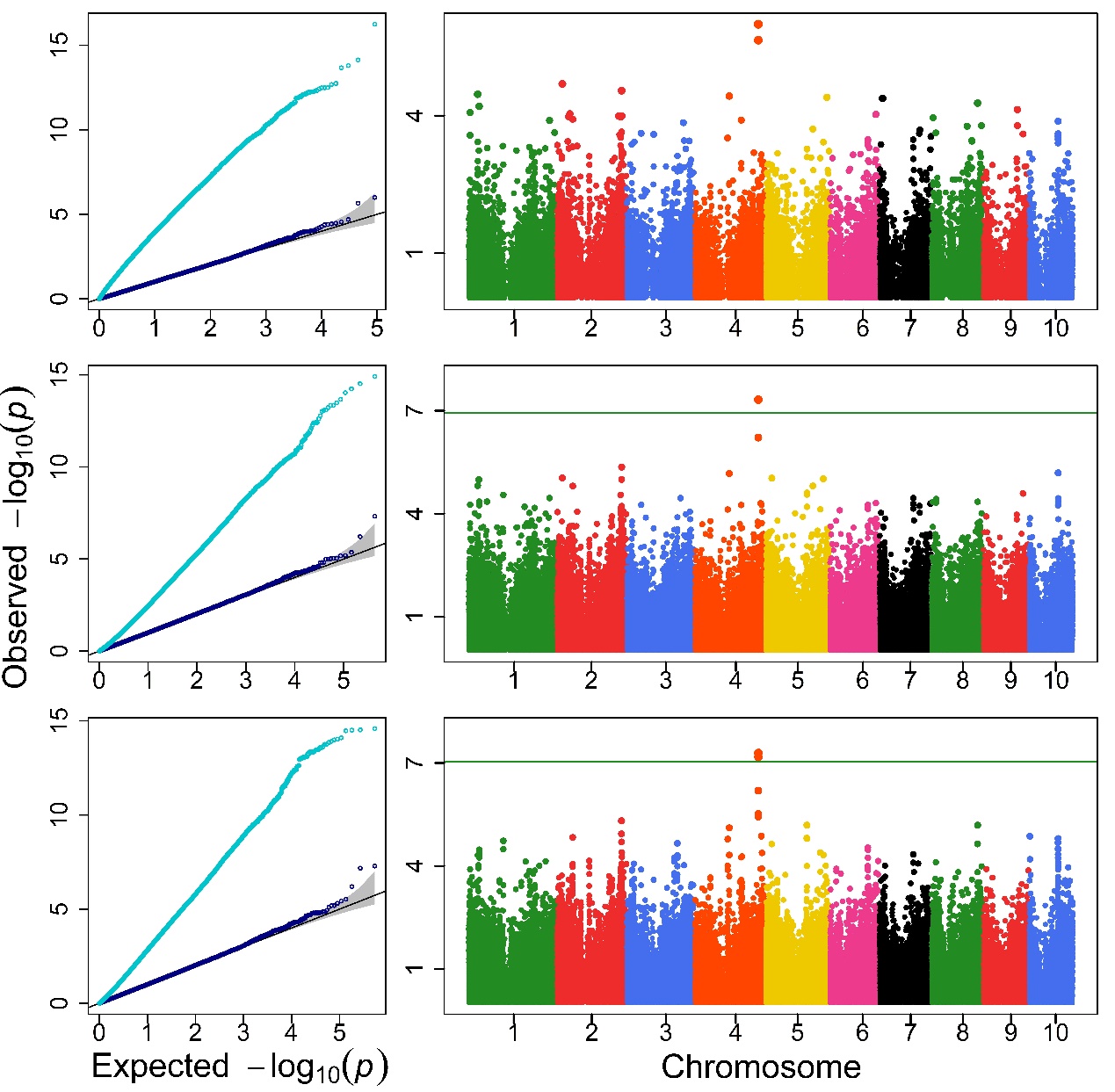


**Figure S1:** QQ and Manhattan plots of three genetic units for LNAE trait. The top, the medium and the bottom are for haplotype blocks, haplotype alleles and SNPs, respectively.

**
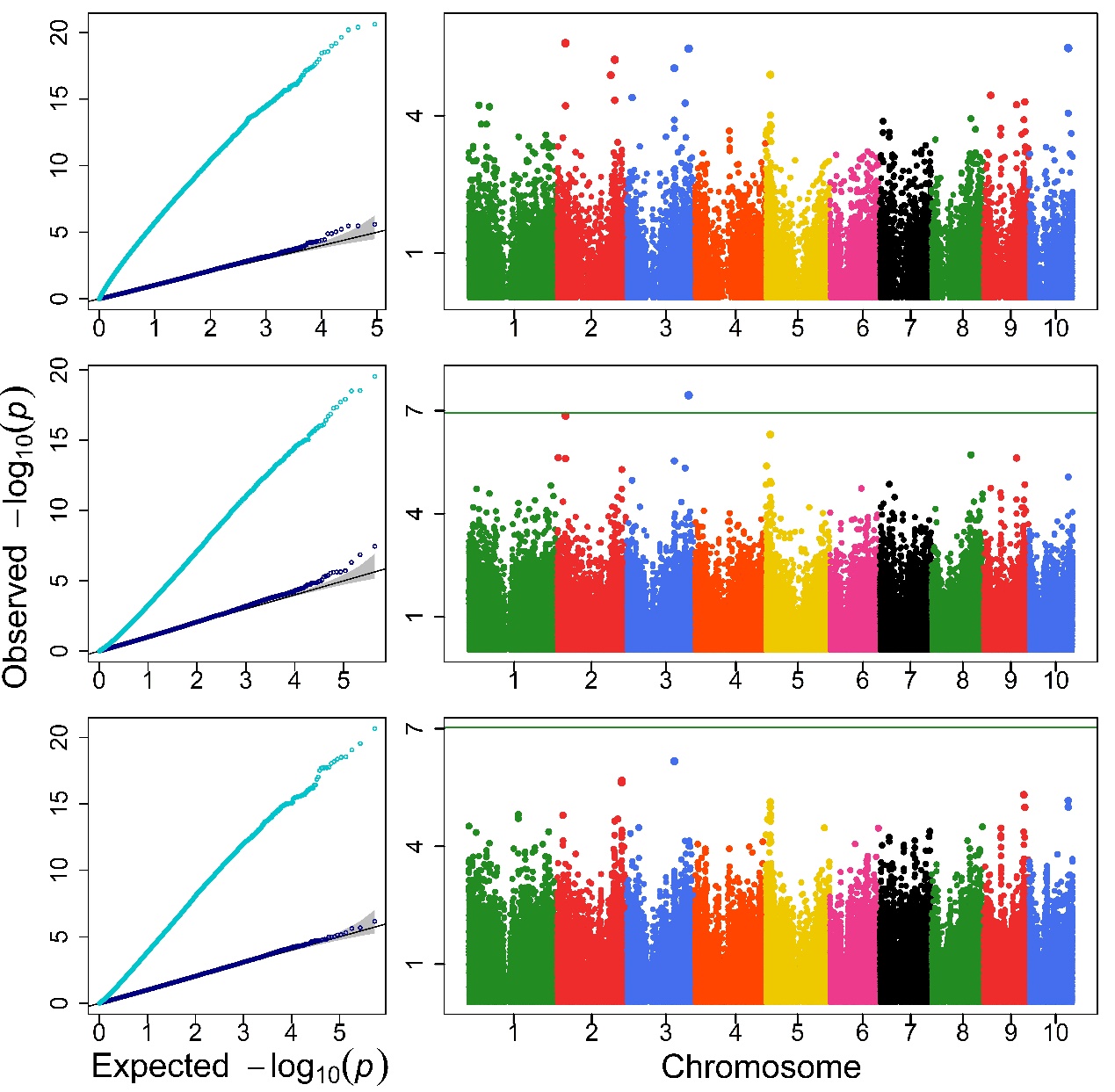
**

**Figure S2:** QQ and Manhattan plots of three genetic units for DTH trait. The top, the medium and the bottom are for haplotype blocks, haplotype alleles and SNPs, respectively.

**
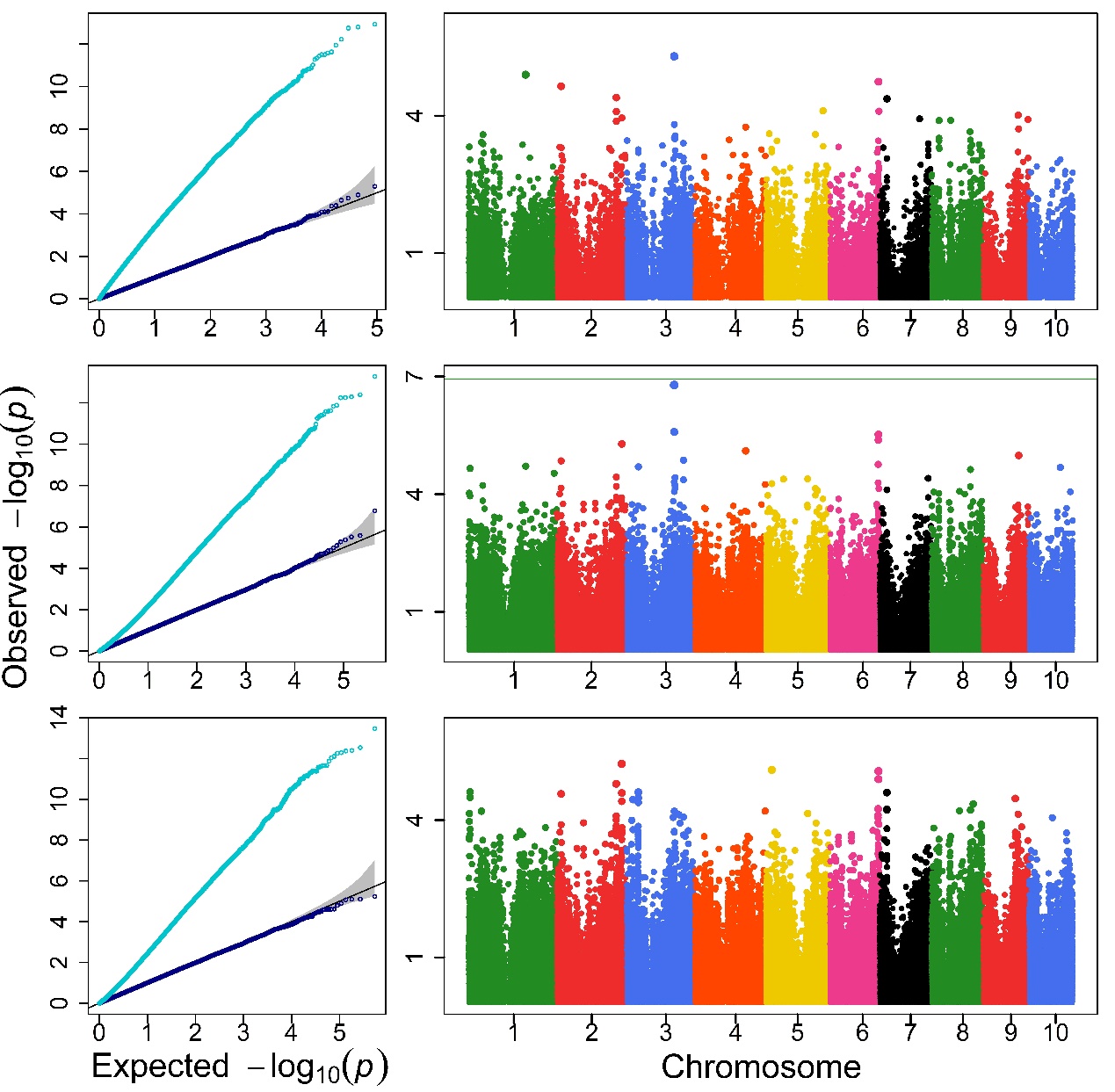
**

**Figure S3:** QQ and Manhattan plots of three genetic units for PH trait. The top, the medium and the bottom are for haplotype blocks, haplotype alleles and SNPs, respectively.

**
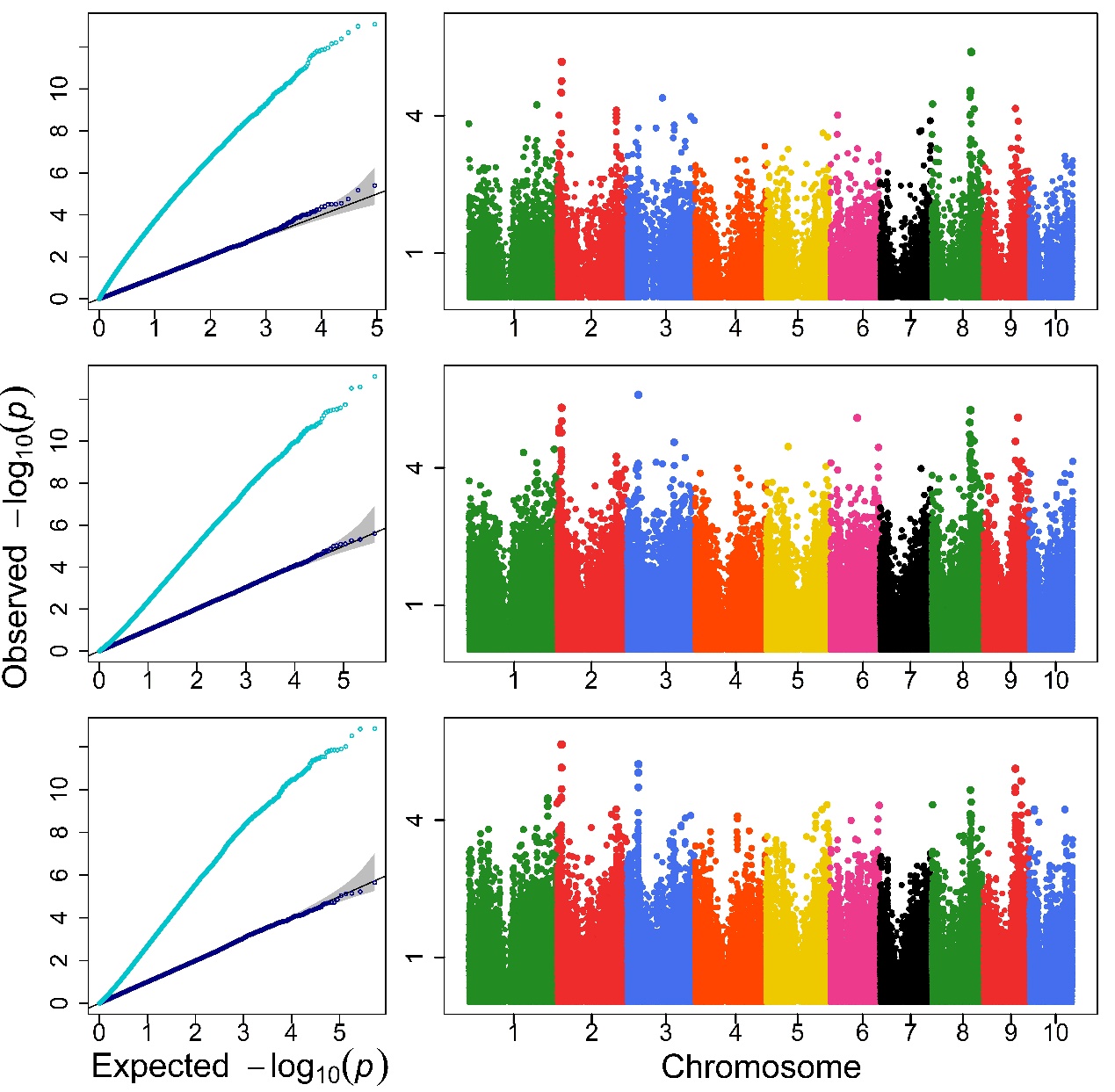
**

**Figure S4:** QQ and Manhattan plots of three genetic units for EH trait. The top, the medium and the bottom are for haplotype blocks, haplotype alleles and SNPs, respectively.

**
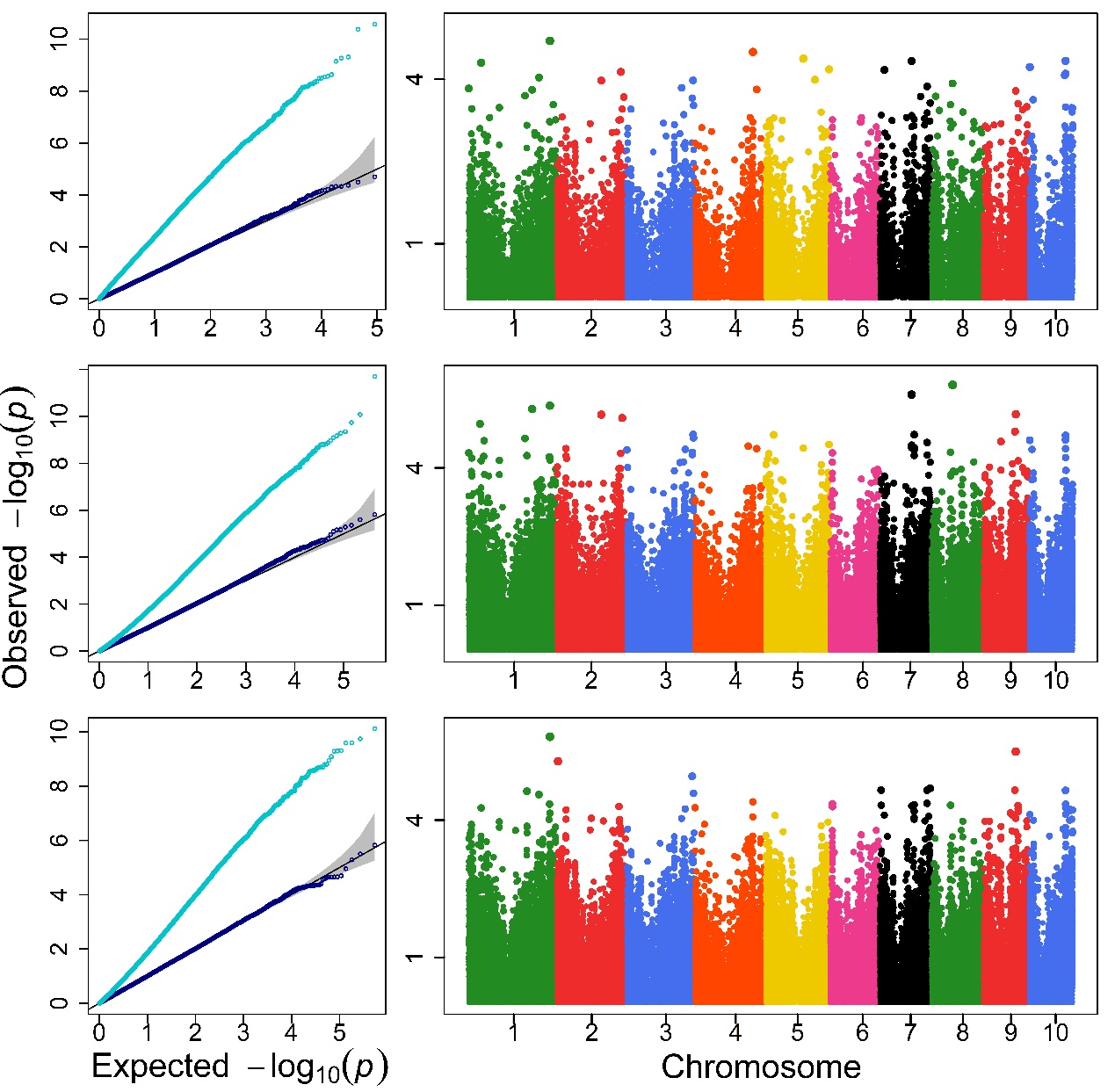
**

**Figure S5:** QQ and Manhattan plots of three genetic units for ELW trait. The top, the medium and the bottom are for haplotype blocks, haplotype alleles and SNPs, respectively.

**
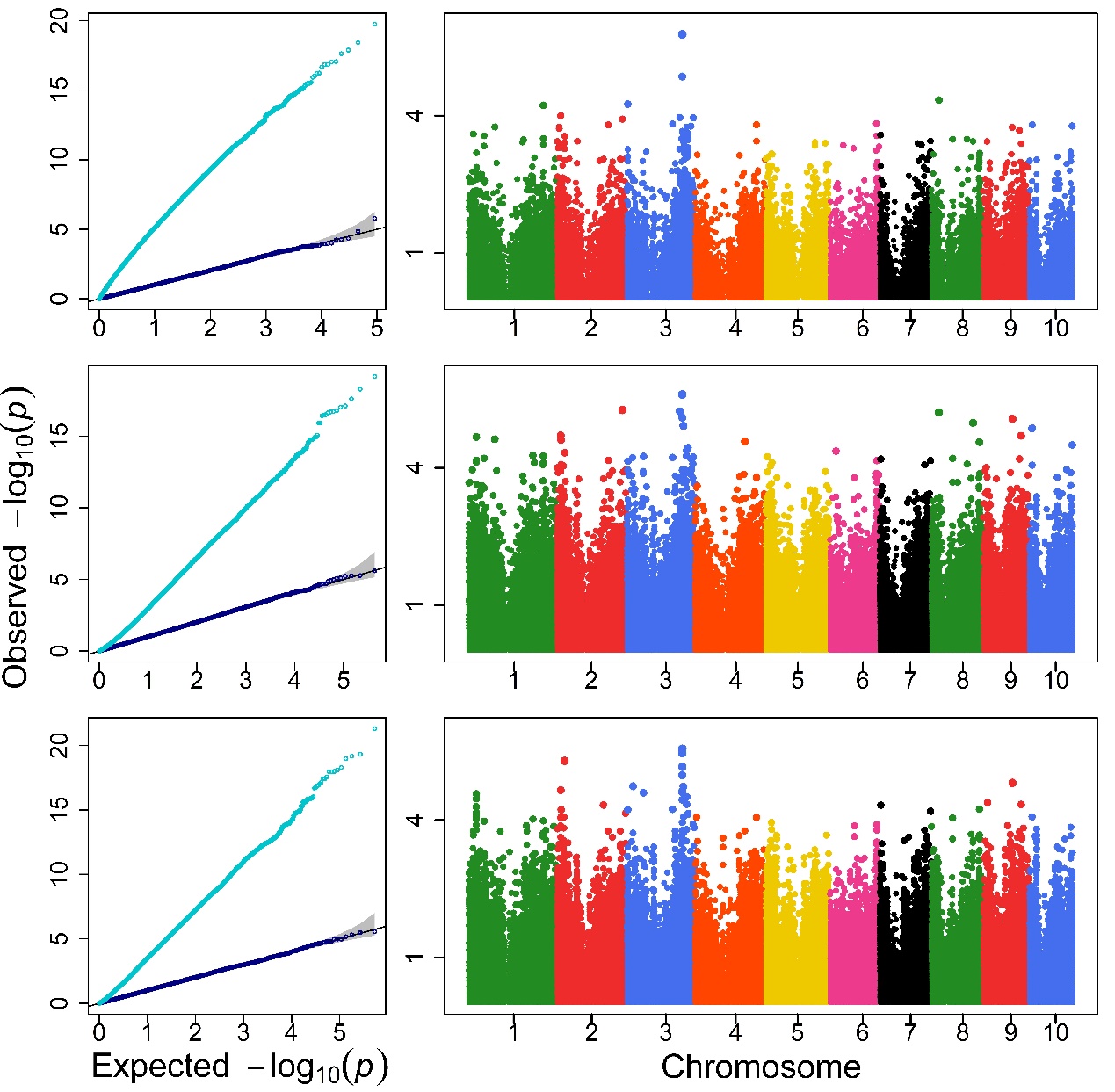
**

**Figure S6:** QQ and Manhattan plots of three genetic units for ELL trait. The top, the medium and the bottom are for haplotype blocks, haplotype alleles and SNPs, respectively.

**
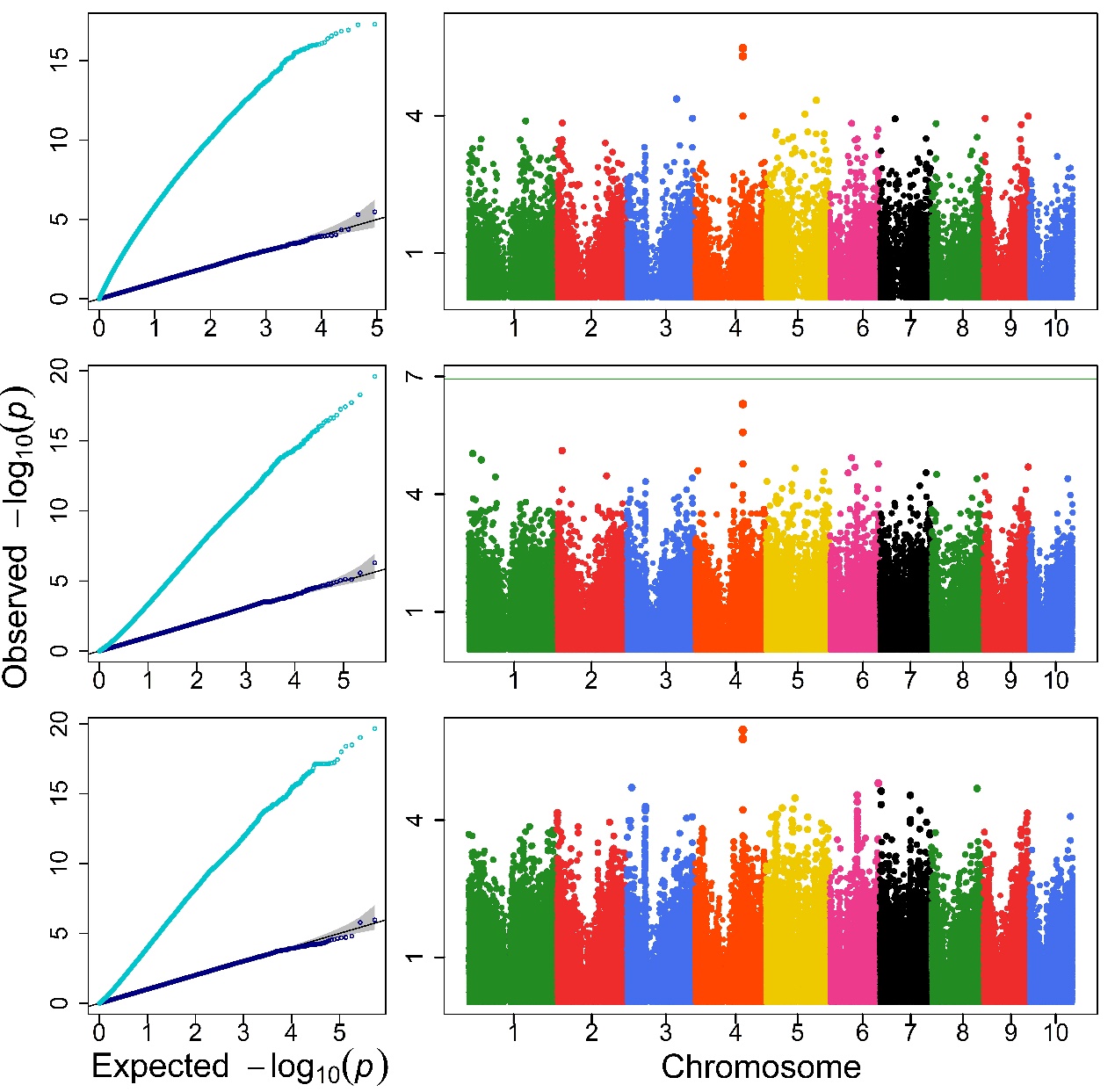
**

**Figure S7:** QQ and Manhattan plots of three genetic units for TBN trait. The top, the medium and the bottom are for haplotype blocks, haplotype alleles and SNPs, respectively.

**
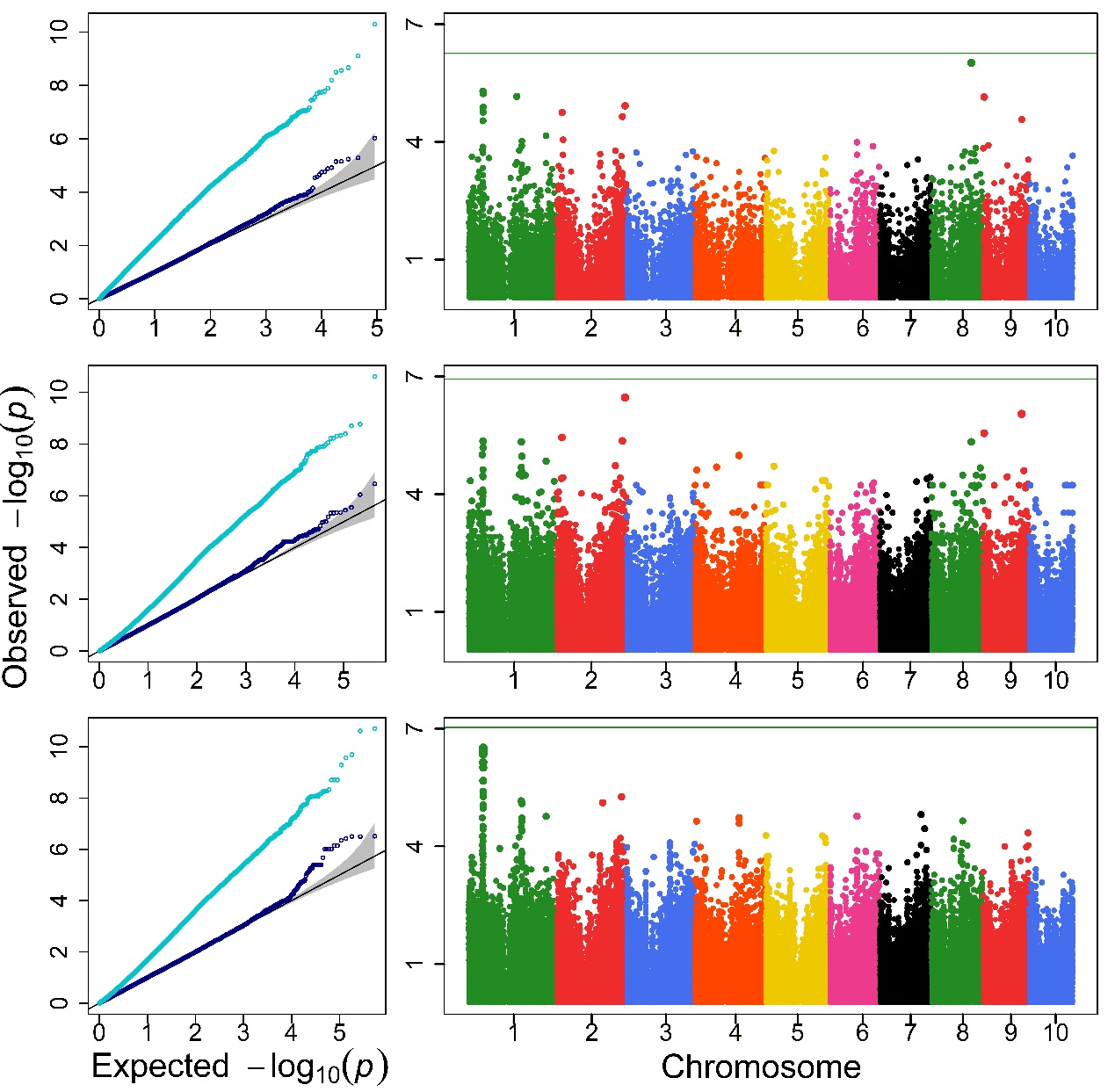
**

**Figure S8:** QQ and Manhattan plots of three genetic units for EL trait. The top, the medium and the bottom are for haplotype blocks, haplotype alleles and SNPs, respectively.

**
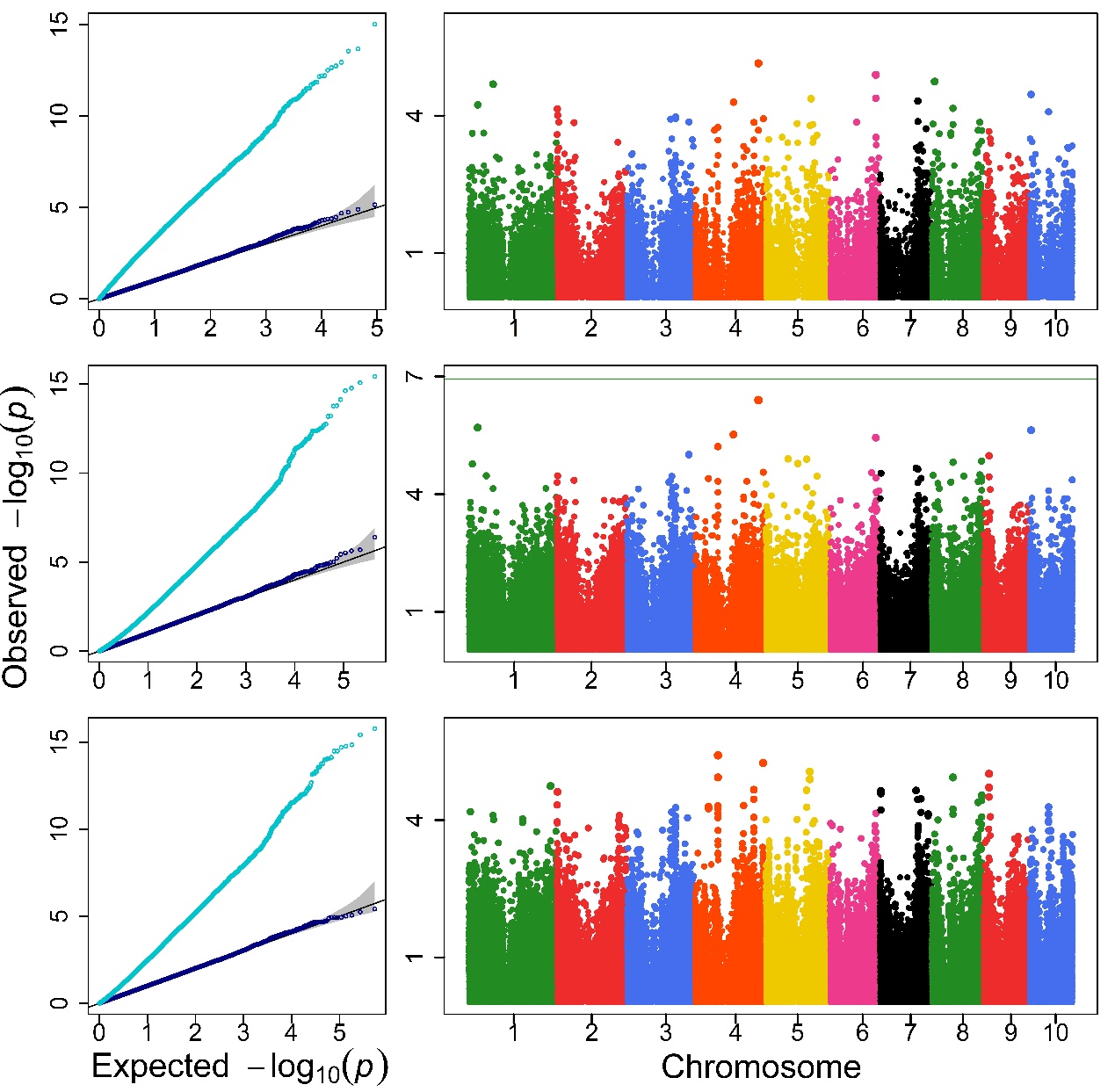
**

**Figure S9:** QQ and Manhattan plots of three genetic units for ED trait. The top, the medium and the bottom are for haplotype blocks, haplotype alleles and SNPs, respectively.

**
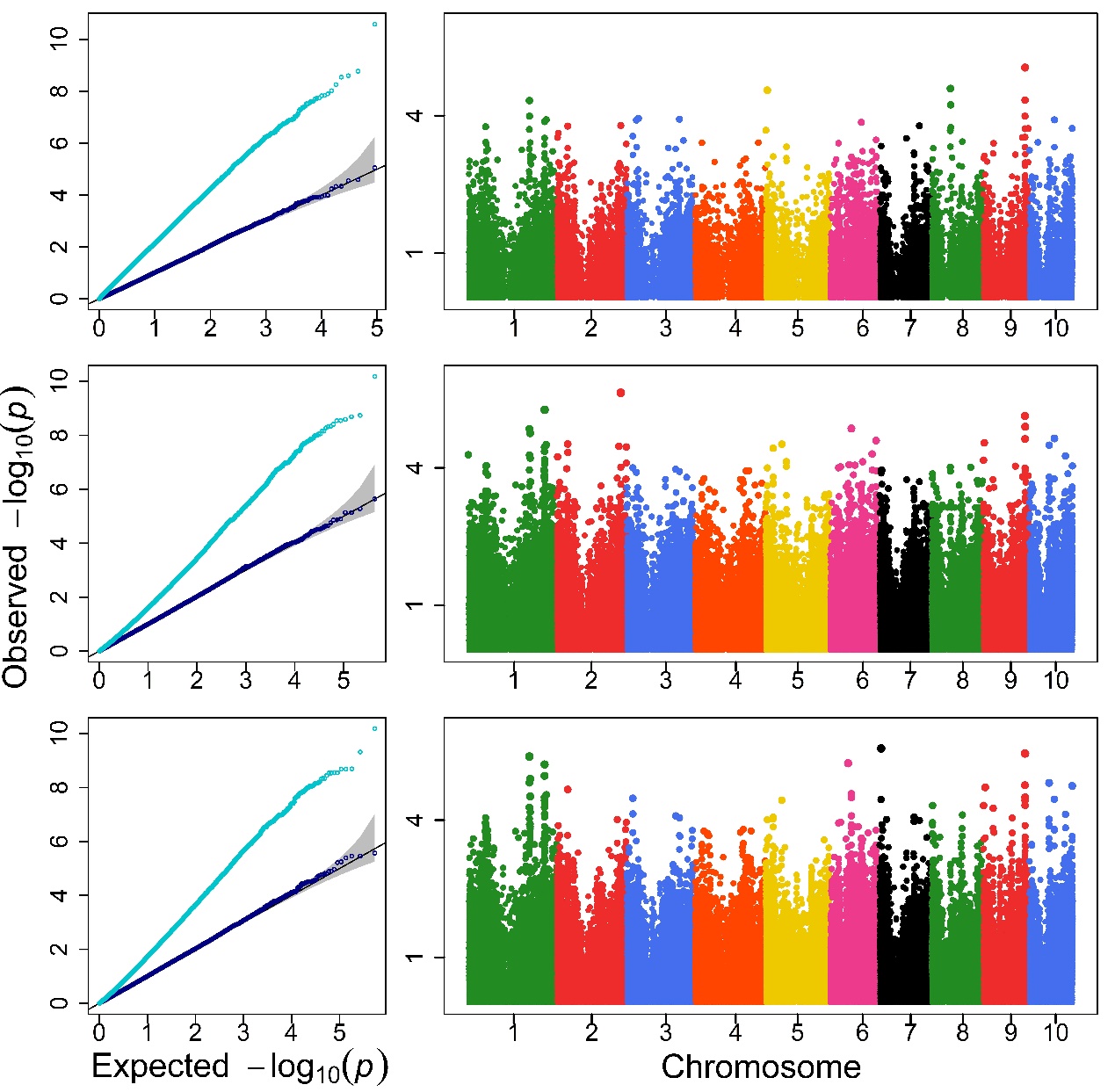
**

**Figure S10:** QQ and Manhattan plots of three genetic units for GW trait. The top, the medium and the bottom are for haplotype blocks, haplotype alleles and SNPs, respectively.

**
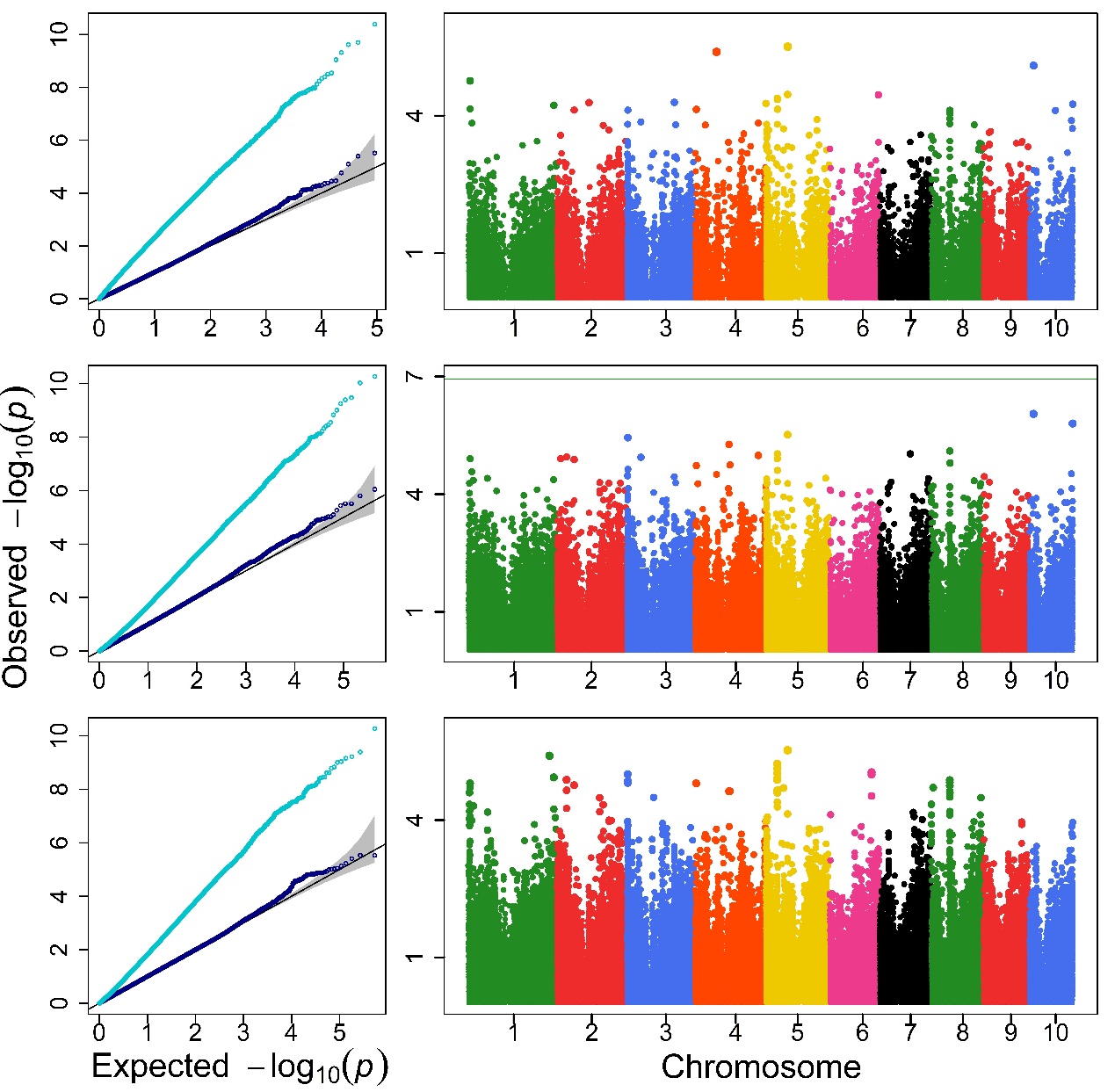
**

**Figure S11:** QQ and Manhattan plots of three genetic units for CW trait. The top, the medium and the bottom are for haplotype blocks, haplotype alleles and SNPs, respectively.

**
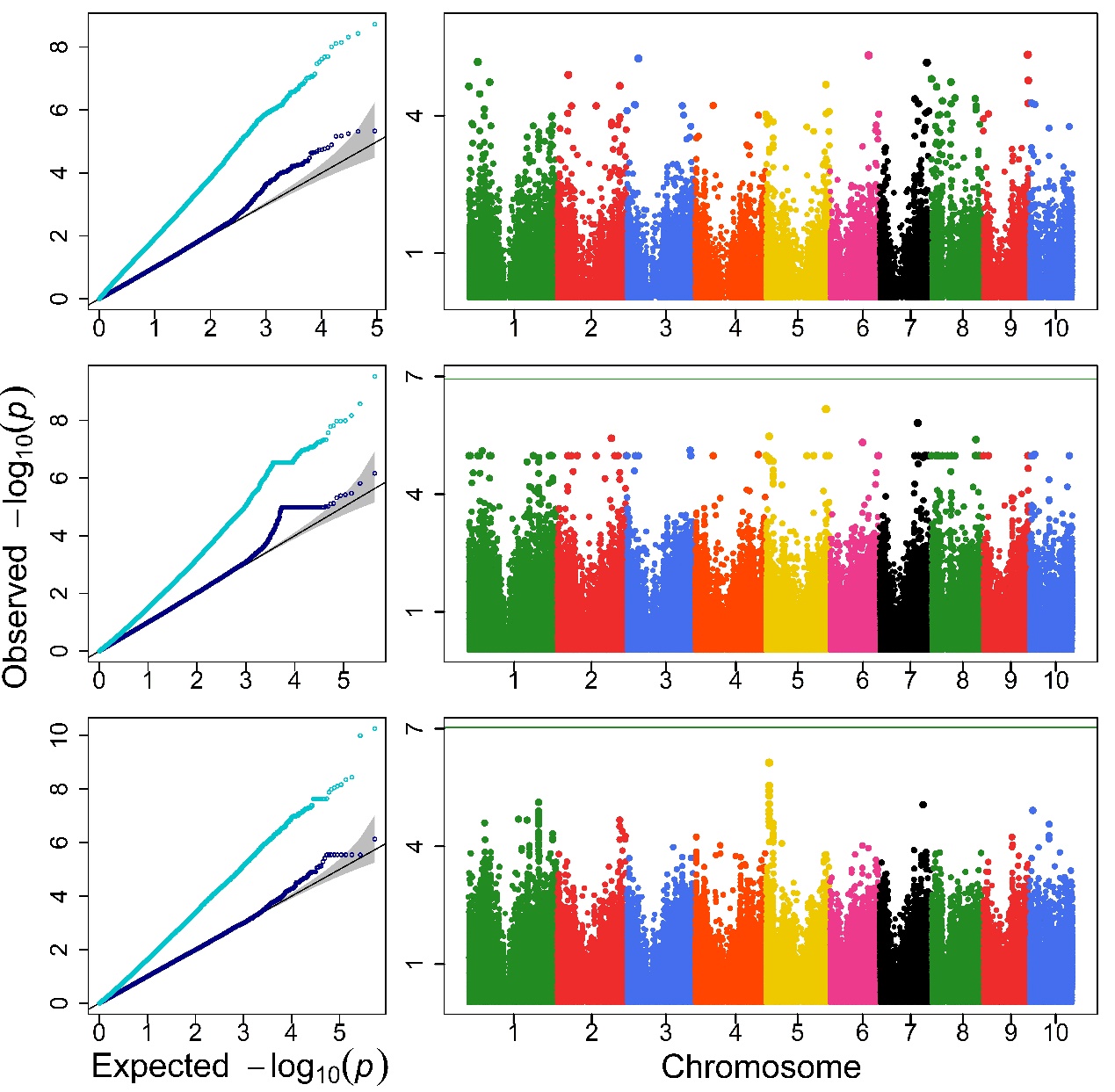
**

**Figure S12:** QQ and Manhattan plots of three genetic units for KW trait. The top, the medium and the bottom are for haplotype blocks, haplotype alleles and SNPs, respectively.

**
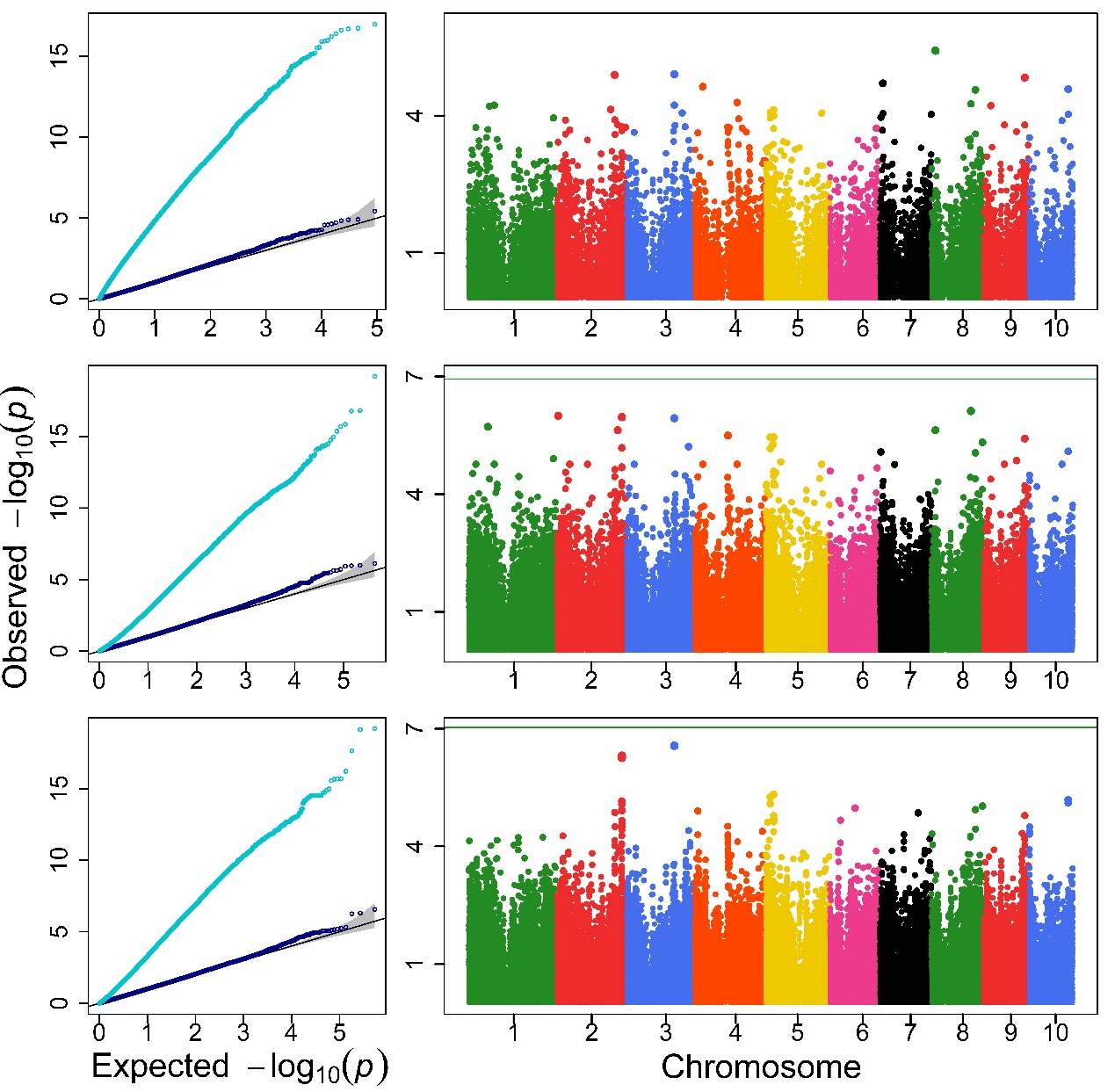
**

**Figure S13:** QQ and Manhattan plots of three genetic units for DTS trait. The top, the medium and the bottom are for haplotype blocks, haplotype alleles and SNPs, respectively.

**
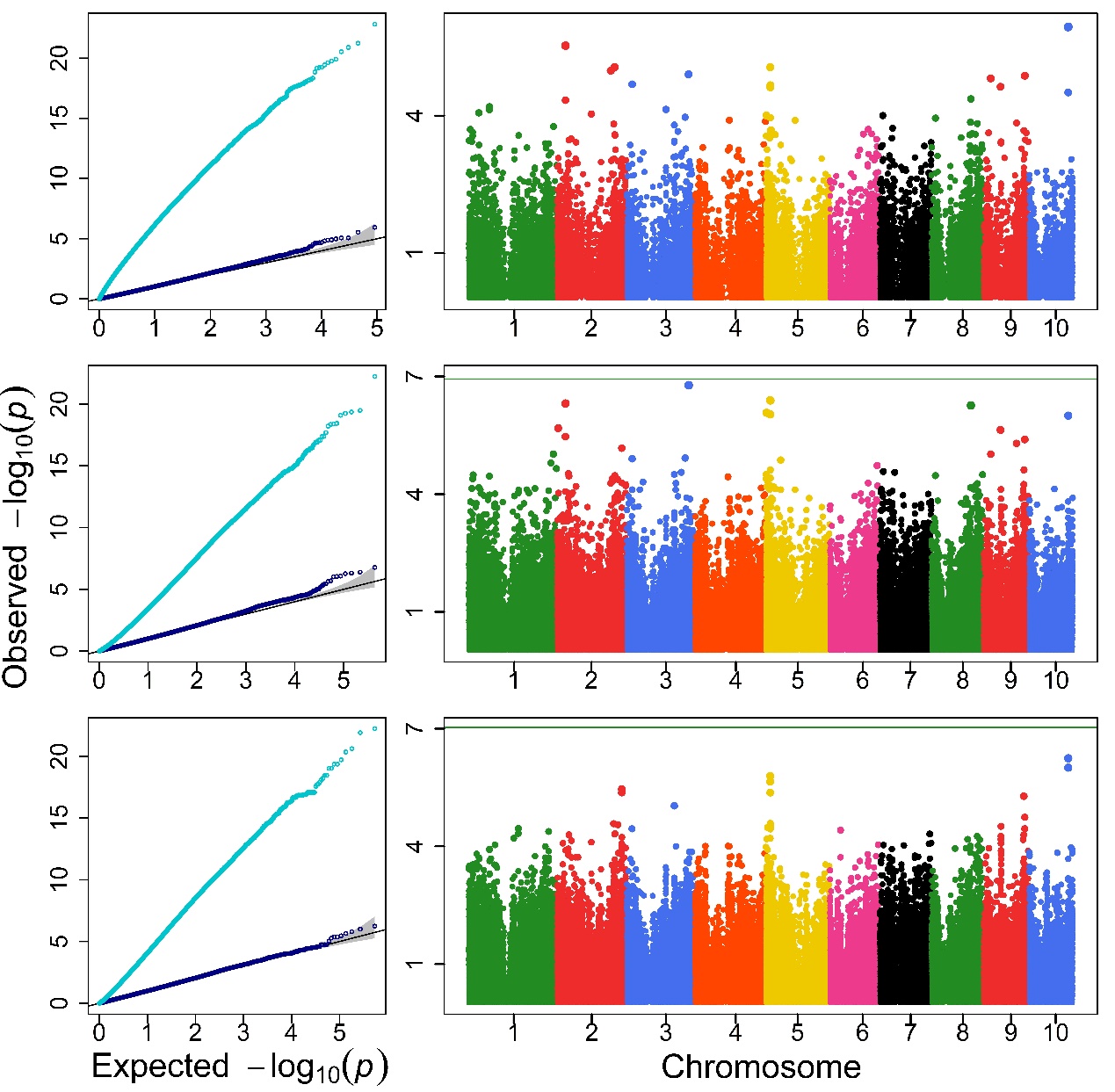
**

**Figure S14:** QQ and Manhattan plots of three genetic units for DTA trait. The top, the medium and the bottom are for haplotype blocks, haplotype alleles and SNPs, respectively.

**Program for Single-Runking**

######################## Subrotunes #################################

GBLUP <- function(ynew,gnew){

nobs <- nrow(gnew)

pre_loglike <- 10^10

ynew <- V050*ynew[,1]

for(i in 1:length(hh)){

gnew1 <- V050[,i]*gnew

fit=fastLmPure(y = ynew[,i], X = gnew1)

eff <- fit$coefficients

resi <- ynew[,i]-gnew1%*%as.matrix(eff)

ve <- sum(resi^2)/(nobs-ncol(gnew)-1)

loglike <- logV0[i]+nobs*log(ve)

if(loglike > pre_loglike){

break

}else{

pre_eff <- eff

h2 <- hh[i]

pre_ve <- ve

pre_loglike <- loglike

}

}

fva <- list(eff=pre_eff,h2=h2,ve=pre_ve,loglike=pre_loglike)

return(c(fva))

}

fastlm_block <- function(i,ynew1,gnew1){

Fs <- c(rep(0,3))

fit <- fastLmPure(y = ynew1[,i], X = gnew1)

y0 <- ynew1[,i]-gnew1[,1]*fit$coefficients[1]

ssy <- sum(y0^2)

resi <- ynew1[,i]-gnew1%*%fit$coefficients

sse <- sum(resi^2)

ssr <- ssy-sse

Fs[3] <- fit$df.residual

ve <- sse/Fs[3]

Fs[2] <- nobs-Fs[3]-1

Fs[1] <- (ssr/Fs[2])/ve

p <- 1-pf(Fs[1],Fs[2],Fs[3])

loglike0 <- logV0[i]+nobs*log(ve)

fastlm_block <- c(fit$coefficients[1],p,loglike0,Fs[1])

}

fast_lmm_block <- function(ynew1,gnew,threshold0){

gnew1 <- V050[,hpos]*gnew

f10 <- fastlm_block(hpos,ynew1,gnew1)

if(f10[2] < threshold0){

pre_eff <- f10[1]

pre_p <- f10[2]

pre_loglike <- f10[3]

pre_F <- f10[4]

for(j in 1:(hpos-1)){

i <- hpos-j

gnew1 <- V050[,i]*gnew

f10 <- fastlm_block(i,ynew1,gnew1)

loglike <- f10[3]

if(loglike > pre_loglike) break

if(i==1) cat("No Heritability!","\n")

pre_loglike <- loglike

pre_eff <- f10[1]

pre_p <- f10[2]

pre_F <- f10[4]

}

hhk <- hh[i+1]

f10 <- c(pre_eff,pre_p,pre_loglike,pre_F)

if(hhk==hh0){

pre_eff <- f10[1]

pre_p <- f10[2]

pre_loglike <- f10[3]

pre_F <- f10[4]

for(j in 1:(length(hh)-hpos)){

i <- hpos+j

gnew1 <- V050[,i]*gnew

f10 <- fastlm_block(i,ynew1,gnew1)

loglike <- f10[3]

if(loglike > pre_loglike) break

if(i==length(hh)) cat("No Solution!","\n")

pre_loglike <- loglike

pre_eff <- f10[1]

pre_p <- f10[2]

pre_F <- f10[4]

}

f10 <- c(pre_eff,pre_p,pre_loglike,pre_F)

}

}

return(f10)

}

QQ_plot <- function(methodname,pValue){

jpeg(file=paste(methodname,".QQ.Plot.jpeg",sep = ""),

width=600*5,height=600*5,res=72*4)

par(mfrow=c(1,1),mar=c(3.1,3.1,0.7,1),oma=c(2.5,2.5,2.5,0),

tcl=-0.5,mgp=c(2.8,1.3,0),lwd = 1.5)

P.values <- as.matrix(pValue)

N=nrow(P.values)

P.values <- as.matrix((P.values)[order(P.values)])

log.P.values <- as.matrix(rev(P.values))

p_value_quantiles <- (1:N)/(N+1)

log.Quantiles <- -log10(p_value_quantiles)

N1=length(log.Quantiles)

c95 <- rep(NA,N1)

c05 <- rep(NA,N1)

for(j in 1:N1){

k=ceiling((10^-log.Quantiles[j])*N)

if(k==0)k=1

c95[j] <- qbeta(0.95,k,N-k+1)

c05[j] <- qbeta(0.05,k,N-k+1)

}

plot(NULL, xlim = c(0,max(log.Quantiles)),

ylim = c(0,max(c(log.P.values,-log10(c05)))),

cex.axis=3.0, cex.lab=2.2, type="l",lty=1, lwd = 5,

axes=TRUE, xlab="", ylab="",col="gray",yaxt ="n",xaxt ="n")

index=length(c95):1

polygon(c(log.Quantiles[index],log.Quantiles),c(-log10(c05)[index],-log10(c95)),

col='gray',border=NA)

abline(a = 0, b = 1, col = "black",lwd=2)

color <- c("navy")

points(log.Quantiles, log.P.values ,col=color,cex=1.1)

x.lim <- max(log.Quantiles)

y.lim <- max(c(log.P.values,-log10(c05)))

axis(1,at=1:ceiling(x.lim),cex.axis=2.3,

labels=c(1:ceiling(max(x.lim))),tick=TRUE,lwd.ticks=3)

axis(2,at=seq(1,ceiling(y.lim),3),cex.axis=2.3,

labels=seq(1,ceiling(y.lim),3),tick=TRUE,lwd.ticks=3)

box()

palette("default")

mtext(expression(Observed~~-log[10](italic(p))),side=2,cex=1.9,outer=TRUE,line=-0.4)

mtext(expression(Expected~~-log[10](italic(p))),side=1,cex=1.9,outer=TRUE)

dev.off()

}

Manh_plot <- function(methodname,manh){

jpeg(file=paste(methodname,".Manhattan.Plot.Genomewise.jpeg",sep = ""),

width=900*5,height=500*5,res=72*4)

par(mfrow=c(1,1),mar=c(3.1,3.1,0.7,1),oma=c(2.5,2.5,2.5,0),

tcl=-0.5,mgp=c(2.8,1.3,0),lwd = 1.5)

manh0 <- manh

cutOff <- 0.05

manh <- matrix(as.numeric(as.matrix(manh0)),nrow(manh0),ncol(manh0))

manh <- manh[manh[,1]!=0,]

numMarker <- nrow(manh)

bonferroniCutOff <- -log10(cutOff/numMarker)

y.lim <- ceiling(max(manh[,3]))

chmtoanalyze <- unique(manh[,1])

nchr <- length(chmtoanalyze)

chrcolor <- c("forestgreen","firebrick2","royalblue2","orangered1",

"gold2","violetred2","black")

plotcolor <- rep(chrcolor,ceiling(nchr/5))

mypch=20

manh <- manh[order(manh[,2]),]

manh <- manh[order(manh[,1]),]

ticks=NULL

lastbase=0

for (i in chmtoanalyze){

index=(manh[,1]==i)

ticks <- c(ticks, lastbase+mean(manh[index,2]))

manh[index,2]=manh[index,2]+lastbase

lastbase=max(manh[index,2])

}

x <- as.numeric(manh[,2])

y <- as.numeric(manh[,3])

z <- as.numeric(manh[,1])

size=1;ratio=10;base=1

themax=ceiling(max(y))

themin=floor(min(y))

wd=((y-themin+base)/(themax-themin+base))*size*ratio

s=size-wd/ratio/2

plot(y~x,xlab="",ylab="" ,ylim=c(0,y.lim),cex.axis=2.1, cex.lab=2.2,col=plotcolor[z],

axes=FALSE,type = "p",pch=mypch,lwd=wd,cex=s+.5,main = "",cex.main=2)

abline(h=bonferroniCutOff,col="forestgreen")

axis(1, at=ticks,cex.axis=2.3,labels=chmtoanalyze,tick=TRUE,lwd.ticks = 3)

axis(2, at=seq(1,floor(y.lim),3),cex.axis=2.3,

labels=seq(1,floor(y.lim),3),tick=TRUE,lwd.ticks = 3)

box()

palette("default")

mtext(expression(Observed~~-log[10](italic(p))),side=2,cex=1.9,outer=TRUE,line=-0.4)

mtext(expression(Chromosome),side=1,cex=1.9,outer=TRUE)

dev.off()

}

############################ Main program ##############################

### Load R packages

library(RcppArmadillo)

library(nleqslv)

### Data input

blup <- read.csv("Phenotype.csv", header=TRUE)

blup[,1] <- as.character(blup[,1])

name_trait <- colnames(blup)[-1]

N_trait <- length(name_trait)

info <- read.table("info.txt", header=TRUE)

info[,1] <- as.numeric(info[,1])

N_marker <- nrow(info)

bonfCut <- -log10(0.05/N_marker)

allchr_matrix <- read.table("allchr_matrix.txt", header=FALSE, sep="\t")

### Calculate frequency of alleles

allelename <- read.table("allelename.txt", header=TRUE)

block <- allelename[ ,1]

fre0 <- table(block)

fre1 <- as.matrix(fre0)

oriname <- rownames(fre1)

fre2 <- cbind(oriname, fre1[ ,1])

blocknew <- block[!duplicated(block)]

Freq <- fre2[blocknew, ]

colnames(Freq) <- c("blockname", "freq")

write.table(Freq, "allele_frequency.txt", row.names=FALSE, col.names=TRUE)

freq <- as.numeric(Freq[ ,2])

timeA <- Sys.time()

###############################################################################

# GWAS Circulation for Traits #

###############################################################################

for (T in 1:N_trait){

name <- name_trait[T]

print(paste("The running trait is ", name, sep=""))

y <- blup[ ,T+1]

g <- allchr_matrix

nobs <- nrow(g)

nmar <- ncol(g)

dp_marker <- NULL

############################################################

# Spectral decomposition #

############################################################

A <- scale(g)

B <- t(A)

G <- A%*%B

G <- G/nmar

g1 <- A

eig <- eigen(G)

sg <- eig$values

ug <- eig$vectors

step <- 1

hh <- seq(0,0.999,0.001*step)

hh <- round(hh,3)

sg <- matrix(rep(sg,length(hh)),nobs)

V <- t(t(sg)*hh/(1-hh)) + 1

logV0 <- apply(log(V),2,sum)

V050 <- 1/sqrt(V)

gnewb0 <- t(ug)%*%as.matrix(rep(1,nobs))

gnw <- t(ug)%*%g1

#### Transform phenotypes

ynew <- t(ug)%*%y

############################################################

# GBLUP(by spectral transformation) #

############################################################

fva0 <- GBLUP(ynew,as.matrix(gnewb0))

hh0 <- fva0$h2

ve0 <- fva0$ve

############################################################

# Single-RunKing #

############################################################

hpos <- which(hh==hh0)

ynew1 <- V050*ynew[,1]

freq <- as.numeric(Freq[ ,2])

dp_marker <- matrix(nrow=N_marker, ncol=5)

### Analysis of genetic units

k1 <- 1

for(j in 1:N_marker){

k2 <- k1+freq[j]-1

if(freq[j]==1){

gnew0 <- cbind(gnewb0, gnw[,c(k1:k2)])

}else{

k3 <- k2-1

gnew0 <- cbind(gnewb0, gnw[,c(k1:k3)])

}

fva <- fast_lmm_block(ynew1, gnew0, 1)

k1 <- k1+freq[j]

dp_marker[j,] <- c(j,fva)

}###j

### GWAS result output

colnames(dp_marker) <- c("Marker","Mean","Pvalue","Loglike","Fvalue")

write.table(dp_marker, paste("GWASresult_", name, ".txt", sep=""), row.names=FALSE, col.names=TRUE)

### QQ and Manhattan plot and significant markers

manh_marker <- data.frame(info[,c(1:3)], dp_marker[,c(2,3)], info[,-c(1:3)])

manh_marker[,5] <- -log10(manh_marker[,5])

QQ_plot(paste("Kfixed_",name,sep=""), manh_marker[ ,5])

Manh_plot(paste("Kfixed_",name,sep=""), manh_marker[ ,c(1,3,5)])

sig <- which(manh_marker[ ,5] > bonfCut)

sig_marker <- manh_marker[sig, ]

write.table(sig_marker, paste("Sig_marker_",name,".txt",sep=""), col.names=TRUE, row.names=FALSE)

}###T

timeB <- Sys.time()

runtime <- timeB-timeA

print(runtime)

########################## End #############################
